# A discovery protein panel for brain predicted age discordance using MRI in neurologically healthy individuals

**DOI:** 10.3389/fcell.2026.1833866

**Published:** 2026-07-09

**Authors:** Jessica M. Gill, Arum Lim, Carrie Esopenko, Sijung Yun, Joseph Yun, Heather E. Dark, John Alice, Kimbra Kenney, James Hentig, Mary Jo Pugh, William C. Walker, David Cifu, Nicola L. de Souza, Emily L. Dennis, Elisabeth A. Wilde

**Affiliations:** 1 Johns Hopkins School of Nursing, Baltimore, MD, United States; 2 Johns Hopkins School of Medicine, Baltimore, MD, United States; 3 Department of Neurology, Uniformed Services University of the Health Sciences, Bethesda, MD, United States; 4 Department of Neurology, University of Utah School of Medicine, Salt Lake City, UT, United States; 5 Department of Neurology, George E. Wahlen Salt Lake City Veterans Affairs Healthcare System, Salt Lake City, UT, United States; 6 Department of Physical Medicine and Rehabilitation, Virginia Commonwealth University School of Medicine, Richmond, VA, United States; 7 Richmond Veterans Affairs Medical Center, Central Virginia VA Health Care System, Richmond, VA, United States

**Keywords:** aging, biomarkers, brain, inflammation, MRI

## Abstract

**Background and Objectives:**

Brain age is a global measure that compares structural brain MRI with large reference datasets. Predicted age deviation (PAD) is the deviation between predicted brain age and chronological age, with positive values indicating advanced aging. Identifying blood-based biomarkers that approximate brain PAD could provide an accessible and cost-effective measure of brain health as an alternative to MRI, but no blood-based biomarkers have yet been identified. This study aimed to investigate novel blood-based biomarkers associated with accelerated PAD using an unbiased proteomics approach to discover new biomarkers.

**Methods:**

This study is a secondary analysis with a cross-sectional case-control design using the LIMBIC-CENC dataset as a discovery approach to understand novel biomarker patterns. Brain age was estimated using brainageR in 137 participants aged ≤40 years with no substantial cognitive deficits or neurological disorders. Cases (n = 76) included individuals with brain age ≥5 years older than chronological age, whereas controls (n = 61) had brain age equal to or younger than chronological age (PAD range: -1.3 to 0; mean = -0.9) and were otherwise matched on demographics and clinical features. Unbiased proteomic profiling of ∼5,400 proteins was performed using the Olink Explore platform. Differential protein expression between groups was assessed using Wilcoxon tests with Benjamini-Hochberg correction. Receiver operating characteristic (ROC) analysis was performed on probabilities derived from generalized linear models (GLMs) to identify optimal protein combinations, prioritizing maximizing both sensitivity and negative predictive value.

**Results:**

Olink analyses identified 418 proteins that were significantly different between groups after multiple-comparison correction. Upregulated proteins in participants with PAD≥5 years included: component inhibitor-nuclear factor kappa-b kinase (CHUK), methenyltetrahydrofolate synthetase domain containing (MTHFSD), and epidermal growth factor (EGF), with log2 fold changes of 1.70–1.80. Insulin-like peptide 3 (INSL3) was the most downregulated protein (log2 fold change −2.27). Enriched pathways involved nuclear factor kappa-b (NF-κB), heat-shock protein, and Wingless/Integrated (Wnt) signaling. Models including 6-7 dysregulated proteins (e.g., CHUK and INSL3) achieved AUCs>0.9, with sensitivities >0.90 and specificities >0.70.

**Discussion:**

These discovery-based findings warrant validation in larger cohorts and suggest potential for blood-based protein panel detection of early, clinically silent, pre-pathological accelerated brain aging changes when interventions may be most effective.

## Introduction

Understanding brain changes before the clinical manifestation of disease (i.e., pre-pathological changes) is gaining focus as a critical pathway to understanding brain health and neurodegenerative disorders (Haugg et al., 2025; Elkana and Beheshti, 2025). Importantly, it enables discovery and development of treatments to attenuate, improve, or reverse the underlying pathology before it produces irreversible clinical symptoms (Haugg et al., 2025; Papouli and Cole, 2025). Brain age measurement originates from the field of biogerontology and provides insights into individual differences in the impact of the aging process (Papouli and Cole, 2025). Advanced brain age has most commonly been reported in middle-aged and older adults and has been linked with declines in cognition and poorer health outcomes in later life (Haugg et al., 2025; Papouli and Cole, 2025). The structure of the brain and its related functions change in both typical aging and disease-associated aging, with individual differences in the nature, progression, and magnitude of these changes (Beydoun et al., 2025). Thus, although people may have the same chronological age, they may have differential aging-related brain changes that are measurable by advanced magnetic resonance imaging (MRI) (Beydoun et al., 2025). Contributing to differences in these individual trajectories may be stable factors such as genetics, sex, and race/ethnicity as well as modifiable factors such as physical activity, sleep, nutrition, environmental exposure, and others, which may further interact with each other to together attenuate or accelerate brain age (Beydoun et al., 2025; Anjum et al., 2025; Namsrai et al., 2025). Therefore, developing improved methods to understand and predict brain age and deviation from chronological age (predicted age deviation [PAD]) prior to the onset of clinical cognitive decline, even during the asymptomatic stage, may enable the development of novel therapies to promote brain health and prevent or reduce cognitive decline (Papouli and Cole, 2025).

Accelerated brain aging has been associated with a number of neurodegenerative disorders, including Parkinson’s disease (PD) (Cai et al., 2025) and Alzheimer’s disease and releated dementias (ADRD) (Papouli and Cole, 2025). Further, within longitudinal studies, there is evidence that brain PAD can predict the development of mild cognitive impairment (MCI) in adults (Elkana and Beheshti, 2025). Even within cohorts of individuals with PD or ADRD, advanced brain PAD is associated with greater cognitive decline and clinical severity, suggesting cognitive decline predictablity (Cai et al., 2025; Pilli et al., 2025).

There is some evidence of fluid biomarkers relating to brain PAD. Plasma neurofilament light chain (NfL), an indicator of neuronal integrity, has been linked to reductions in brain volume in cognitively unimpaired individuals (Coors et al., 2025). Further, phosportylated tau (p-tau) was linked with reduced cortical thickness and increased brain PAD in a cognitively healthy community cohort (Sim et al., 2025). Lastly, these established proteins, including amyloid β, also relate to a greater risk of ADRD, ADRD progression, and MCI conversion to ADRD within older cohorts (Pahlke et al., 2025). Still, in younger populations without ADRD or MCI, these same proteins are not as well understood in relation to brain PAD. Since PAD is a complex and composite measure of brain health (Casanova et al., 2024), we used a high-throughput proteomic discovery method (Olink Explore) in an ongoing longitudinal sample of veterans and evaluated brain PAD as measured by brainageR (https://github.com/james-cole/brainageR) to discover proteins and pathways underlying these brain PAD differences, to develop novel lines of preliminary inquiry.

## Methods

### Study design and participants

This secondary analysis utilized a cross-sectional subset of data collected under the Long-term Impact of Military-related Brain Injury Consortium—Chronic Effects of Neurotrauma Consortium (LIMBIC-CENC) prospective longitudinal study (PLS), which enrolled service members and veterans with previous combat deployment(s) (Cifu, 2022/04; Walker et al., 2016).

In the overall LIMBIC-CENC PLS, inclusion criteria were: (a) adult age >18 years and (b) a history of United States military combat deployment (Vogt et al., 2013). Exclusions were: (a) history of moderate or severe traumatic brai injury (TBI) as defined as initial Glasgow Coma Scale < 13, loss of consciousness duration > 30 min, post-traumatic amnesia duration > 24 h, or traumatic intracranial lesion on head computed tomography (CT); (b) a history of major neurologic or psychiatric disorder such as stroke, spinal cord injury, or schizophrenia (Walker et al., 2016).

For this secondary analysis, we developed a cross-sectional case-control design which included LIMBIC-CENC participants with a MRI and plasma, who were below 40 years, had no major neurological disease (PD, AD, MCI, and multiple sclerosis), and had moderate to high cognitive performance based on latent profiles as described in methods published previously (de Souza et al., 2024). The cases were defined as those with a brain PAD≥5 years and the control group had a brain PAD between 0 and -1.5 (both compared to chronological age). Cases and controls were then matched on the following clinical and demographic features: (a) sympotom levels of PTSD assessed using the post-traumatic stress disorder symptom checklist for DSM-5 (PCL-5); (b) symptom levels of depression assessed using Patient Health Questionnaire-9 (PHQ-9); (c) history of TBIs; (d) sex; (e) age; (f) race; and (g) ethnicity to allow for a more approximate comparison.

### Standard protocol approvals, registrations, and patient consents

The LIMBIC-CENC study received regulatory and ethical approvals from all local and central Institutional Review Boards (IRB) and facilities, and written informed consent was obtained from participants prior to any procedures were performed, and secondary review at Johns Hopkins University IRB (IRB00400584).

## Measurements

### MRI acquisition

Three-dimensional T1-weighted images were acquired at all sites using protocols recommended by the Alzheimer Disease Neuroimaging Initiative and consistent with other large TBI-based consortia. All sites followed established quality control procedures and audits to ensure data quality and consistency.

#### Brain predicted age deviation

We applied the pre-trained BrainageR v2.1 model, 28,765,056 which uses a Gaussian Process Regression with an RBF kernel and was trained on n = 3,377 healthy individuals. The model is distributed as a pre-computed object, and our prediction pipeline simply applies this published model to new data without any hyperparameter tuning or nested cross-validation on our part. The model’s published performance on held-out test data (R^2^ = 0.946, MAE = 3.93 years) and independent validation on the CamCAN dataset (r = 0.947, MAE = 4.90 years) provides confidence in its generalizability. 28,765,056 with a model similar to those described previously (Cole et al., 2015). T1-weighted MRIs from 3,377 individuals in seven publicly available datasets were used to train the original brainageR model, including individuals 18–92 years old from the United States, United Kingdom, Australia, and China. Briefly, T1-weighted MRIs were segmented into gray matter, white matter, and cerebrospinal fluid using SPM12 (https://www.fil.ion.ucl.ac.uk/spm/software/spm12/) and spatially normalized. The resulting images were vectorized and subjected to principal components analysis (using R *prcomp* https://cran.r-project.org). Components explaining the top 80% of variance were retained, resulting in 435 components.

The brainageR model was trained using mean absolute error (MAE) of y (i.e., age) as the loss function. The kernel used was the radial basis function. All other GPR parameters were set as the default in kernlab (https://cran.r-project.org/web/packages/kernlab/kernlab.pdf), except for the initial noise variance, which was determined using nested cross-validation for hyperparameter tuning (comparing values of: 0.001, 0.01, 0.1, 1, 10), which gave 0.001 as the optimal value that was used for final training. The model’s R^2^ (0.67) between predicted and chronological brain age, indicating reasonable model performance.

Processing of the LIMBIC-CENC data was the same as described above for the larger sample, with raw T1-weighted MRIs segmented, normalized, vectorized, and the rotation matrix from the training dataset applied to yield 435 components for each participant. The resulting components were used to predict brain age using *kernlab*, and tissue segmentations were visually checked for quality.

### Blood sample acquisition

Peripheral whole blood was collected using an EDTA tube during the baseline comprehensive evaluation. Samples were processed into plasma and aliquoted, then frozen at −20 °C at the study sites. Frozen samples were shipped to the LIMBIC-CENC Biorepository and stored at −80 °C until retrieved for analysis.

### Unbiased biomarker analysis

Proteomic profiling was performed using the Olink Explore platform, a high-throughput Proximity Extension Assay (PEA)-based technology enabling multiplex quantification of approximately 5,400 protein biomarkers. The assay relies on pairs of antibody binding and DNA-based detection, providing high specificity and sensitivity in complex biological matrices, such as plasma or serum. For this study, plasma samples were aliquoted into pre-assigned wells and transferred to sample source plates using a F.A.S.T.® instrument, followed by automated dilution with the SPTlabtech Dragonfly® instrument. During incubation, PEA™ probe pairs bound to their target proteins, allowing the attached DNA oligonucleotides to come into proximity. Following incubation, hybridized oligonucleotides were extended to generate unique DNA barcodes using a DNA polymerase, which were subsequently amplified by a Polymerase Chain Reaction (PCR). Sample-specific indexes were added to enable pooling of amplicons, producing libraries containing assay-specific barcodes, sample indexes, and Illumina sequencing adapters P5 and P7 Adapters and Sequencing Primer Binding Site Rd1SPLibrary purification and samples were then pooled into the final Explore HT library and sent for sequencing by NGS using Illumina platform. The relative concentration of each biomarker, based on matched counts was calculated using the Olink NPX Explore HT software. Protein expression was normalized using intensity-based normalization to generate Normalized Protein eXpression (NPX) values.

## Statistical analysis

### Group differences in blood biomarkers

We used the Wilcoxon test with the olink_one_non_parametric function from the OlinkAnalyze package (v4.0.1) in R (v4.4.0). The Benjamini-Hochberg’s False Discovery Rate (FDR) method was used to account for multiple testing, which is widely used in proteomic discovery.34,589,181 From this analyses we identify the proteins with the lowest adjusted p-values for progression in the analytical pipeline to develop models.

### Area under curve (AUC) of receiver operating characteristic (ROC) and optimal cutoff determination for sensitivity and specificity

Generalized Linear Models (GLMs) were employed to identify optimal combinations of biomarkers for classification tasks. The GLM was trained using protein expression data as predictors and the corresponding grouping data as the response variable, enabling the model to establish relationships between biomarker expression and group classifications. After training, the predictions generated by the GLM underwent an additional area under the receiver operating characteristic curve (AUC) analysis to determine optimal cutoffs. These cutoffs were used to assess key performance metrics: Sensitivity, Specificity, Precision, and Negative Predictive Value (NPV).

### Ingenuity pathway analysis

Ingenuity pathway analysis (IPA) was used to display proteomic pathways and networks. The input for IPA include proteins differentially expressed between the two groups with adjusted p-value <0.05 and log2 Fold Change (log2FC) greater or less than 1 which were uploaded to IPA for Core Analysis (QIAGEN Inc., https://digitalinsights.qiagen.com/IPA). IPA then compares protein abundance between conditions (e.g., high PAD vs. low or no PAD) to identify differentially expressed proteins. Protein networks were created with nodes nd connections based on references from literature, textbooks, or canonical data in the QIAGEN knowledge base of human orthologs. Next, IPA maps proteins to known signaling pathways and generates figures that describe biological functions, and allow for visualization of interactions, upstream regulators, and downstream effects in interactive pathway maps that relate to differential protein abundance in the cases vs. control comparison. The color and intensity of the nodes indicate the level of dysregulation (red for upregulation, green for downregulation) and predicted activation status (orange for activated, blue for inhibited). The relationships between nodes are represented by solid lines (direct) or dashed lines (indirect).

### Data availability

Anonymized data not published within this article will be made available by request from any qualified investigator.

## Results

### Demographics and PAD grouping features

This study’s final sample consisted of 137 participants, described in Table 1. The advanced aging group (n = 76; PAD≥5 years compared to chronological age) and the control group (n = 61; brain PAD≤0 with a range of −1.3–0, and a mean of −0.9) were similar in all demographic and clinical features examined. The mean absolute error was 4.4 years; however, we note that this value is essentially equivalent to the mean PAD across the sample (−4.4 years).

**TABLE 1:** Sample characteristics.

Cases	PAD ≥5 years	Controls	Group differences
	(N = 76)	(N = 61)	p-value
	Mean ± SD or N (%)		
Age (years)	30.9 ± 10.9	31.3 ± 8.6	0.29
Sex	​	​	0.18
Male	52 (68.42)	47 (77.05)	​
Race/ethnicity	​	​	0.48
Non-Hispanic white	50 (65.79)	44 (72.13)	​
Non-Hispanic black	11 (14.45)	9 (14.75)	​
Hispanic	14 (18.42)	7 (11.48)	​
Non-Hispanic other	1 (1.31)	1 (1.64)	​
Education	​	​	0.43
High school or below	41 (53.95)	31 (50.81)	​
Some college	21 (27.63)	23 (37.70)	​
College graduate	14 (18.42)	7 (11.48)	​
Marital status	​	​	0.46
Married	52 (68.42)	37 (60.66)	​
Unmarried	24 (31.58)	24 (39.34)	​
Years in military service	11.8 ± 7.8	10.4 ± 5.4	0.25
Years in warzone	2.0 ± 1.6	2.4 ± 2.2	0.70
Combat severity	42.8 ± 14.8	41.3 ± 16.4	0.08
PTSD severity	17.09 ± 10.21	18.09 ± 12.92	0.19

PAD: Predicted age deviation, PTSD: Post-traumatic stress disorder.

Combat severity was assessed using the deployment risk and resilience inventory-2 (DRRI-2) combat experience total score. PTSD, severity was measured using the post-traumatic stress disorder symptom checklist for DSM-5 (PCL-5).

### Discovery of dysregulated proteins in advanced brain PAD cases

We found that 418 proteins were significantly (at least two-fold change) different in PAD≥5 cases compared to controls, after adjusting for multiple comparisons. Table 2 provides a summary of the 20 proteins with the most significant adjusted p-values and fold changes (adjusted p-value <0.05 and |log2FC|>1), listed in order of lowest adjusted p-value. Most proteins were upregulated (19 out of 20); the highest fold differences in PAD≥5 cases compared to controls were in component inhibitor-nuclear factor kappa-b kinase (CHUK), methenyltetrahydrofolate synthetase domain containing (MTHFSD), and epidermal growth factor (EGF), with log2FC ranging from 1.69 to 1.80. Insulin like 3 (INSL3) was the only downregulated protein among the top 20 proteins, with a log2FC of −2.27.

**TABLE 2:** Dysregulated proteins in PAD≥5 cases compared to controls.

Protein	Primary functions	Log2Fold change	Log2 Average PAD ≥5	Log2 Average controls	Adjusted p-value*
CHUK (component of inhibitor of nuclear factor kappa B kinase complex)	NF-κB activation, inflammation	1.8025	1.146	−0.6565	9.61E-05
MPIG6B (megakaryocyte and platelet inhibitory receptor G6b)	Platelet inhibition, Hemostasis	1.4116	0.9289	−0.4827	9.61E-05
ASAP3 (ArfGAP with SH3 domain, ankyrin repeat and PH domain 3)	Cell migration, Cytoskeleton remodeling	1.0659	0.7805	−0.2854	1.76E-04
INSL3 (insulin like 3)	Reproductive hormone, Bone Metabolism	−2.2707	−2.9074	−0.6366	2.36E-04
FEZ2 (fasciculation and elongation protein zeta 2)	Axon elongation, neuronal transport	1.1726	0.6462	−0.5264	2.55E-04
PMM2 (Phosphomannomutase 2)	Glycosylation, Metabolism	1.2681	0.9232	−0.3448	2.55E-04
EGF (epidermal growth factor)	Cell proliferation, growth signaling	1.6975	1.1607	−0.5367	2.57E-04
IKBKG (inhibitor of nuclear factor kappa B kinase regulatory subunit gamma)	NF-κB regulation, immune signaling	1.3841	0.8904	−0.4937	2.57E-04
NEDD1 (NEDD1 gamma-tubulin ring complex targeting factor)	Microtubule nucleation, Mitosis	1.2732	0.8914	−0.3817	2.57E-04
RNF41 (ring finger protein 41)	Ubiquitination, receptor degradation	1.0354	0.7841	−0.2512	2.57E-04
PZP (PZP alpha-2-Macroglobulin like)	Protease inhibition	1.0681	0.9560	−0.1120	2.75E-04
PRKD2 (protein kinase D2)	Signal transduction, cell survival	1.0407	0.6751	−0.3656	2.76E-04
ARHGEF12 (rho guanine nucleotide exchange factor 12)	RhoA activation, Cytoskeleton dynamics	1.6091	0.9273	−0.6817	2.81E-04
BACH1 (BACH transcriptional regulator 1)	Transcriptional repression, oxidative stress	1.1249	0.9836	−0.1412	2.81E-04
DUSP19 (Dual specificity phosphatase 19)	MAPK regulation, stress response	1.0791	0.8585	−0.2205	2.81E-04
GEMIN2 (gem nuclear organelle associated protein 2)	snRNP assembly, RNA splicing	1.3622	0.9552	−0.4069	2.81E-04
MTHFSD (methenyltetrahydrofolate synthetase domain containing)	One-carbon metabolism, folate pathway	1.7831	1.1656	−0.6174	2.81E-04
SULT1A1 (Sulfotransferase family 1A member 1)	Sulfation, Detoxication	1.4719	1.0647	−0.4071	2.81E-04
THTPA (thiamine triphosphatase)	Thiamine metabolism, Phosphorate regulation	1.2043	0.8852	−0.3190	2.81E-04
TSC1 (TSC complex subunit 1)	mTOR inhibition, tumor suppression	1.2555	0.5876	−0.6678	2.81E-04

* Ordered by adjusted p-values.

### Discovery panels of proteins for distinguishing advanced brain PAD cases

**FIGURE 1:**
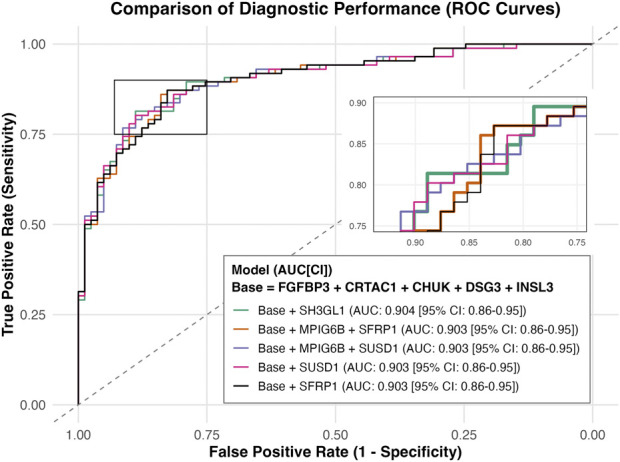
ROC curve for AUC models CI: Confidence Interval; ROC: Receiver operating characteristic; AUC: Area under the curve.

Table 3 provides information related to the combined value of proteins in distinguishing PAD≥5 cases from controls. The top performing panel includes the following proteins: Fibroblast Growth Factor Binding Protein 3 (FGFBP3) + Cartilage Acidic Protein 1 (CRTAC1) + Component of Inhibitor of nuclear factor kappa-b kinase complex (CHUK) + desmoglein 3 (DSG3) + insulin like 3 (INSL3) + SH3 domain containing GRB2 like 1, endophilin A2SH3GL1 (SH3GL1), resulting in an AUC of 0.9035 and specificity and sensitivity of 0.9135 and 0.7326, respectively. Additionally, in model 2, the removal of SH3GL1 and addition of megakaryocyte and platelet inhibitory receptor G6b (MPIG6B) and secreted frizzled related protein 1 (SFRP1) result in a model with slightly greater sensitivity (0.9259 vs. 0.9135), but slightly lower specificity (0.7093 vs. 0.7326), suggesting the differing proteins combinations can distinguish PAD≥5 from controls. Also, additional opportunities for model development based on desired explanatory outcomes using different combinations of these proteins is possible. These models are depicted in Figure 1 in ROC curves.

**TABLE 3:** Panels of Proteins for Distinguishing PAD≥5 cases from Controls.

Proteins panel	AUC	Sensitivity	Specificity	Precision	NPV	FPR	FNR
FGFBP3 + CRTAC1 + CHUK + DSG3 + INSL3 + SH3GL1	0.9035	0.9135	0.7326	0.7629	0.9076	0.2674	0.0864
FGFBP3 + CRTAC1 + CHUK + MPIG6B + DSG3 + INSL3 + SFRP1	0.9034	0.9259	0.7093	0.7565	0.9105	0.2907	0.0741
FGFBP3 + CRTAC1 + CHUK + MPIG6B + DSG3 + INSL3 + SUSD1	0.9034	0.9136	0.7674	0.7872	0.9041	0.2326	0.0864
FGFBP3 + CRTAC1 + CHUK + DSG3 + INSL3 + SUSD1	0.9023	0.9136	0.7442	0.7708	0.9014	0.2558	0.0864
FGFBP3 + CRTAC1 + CHUK + DSG3 + INSL3 + SFRP1	0.9025	0.9259	0.6977	0.7426	0.9091	0.3023	0.0741

AUC: Area under the curve, NPV: Negative predictive value, FPR: False positive rate, FNR: False negative rate.

### Discovery protein networks by ingenuity pathway analysis (IPA)

**FIGURE 2:**
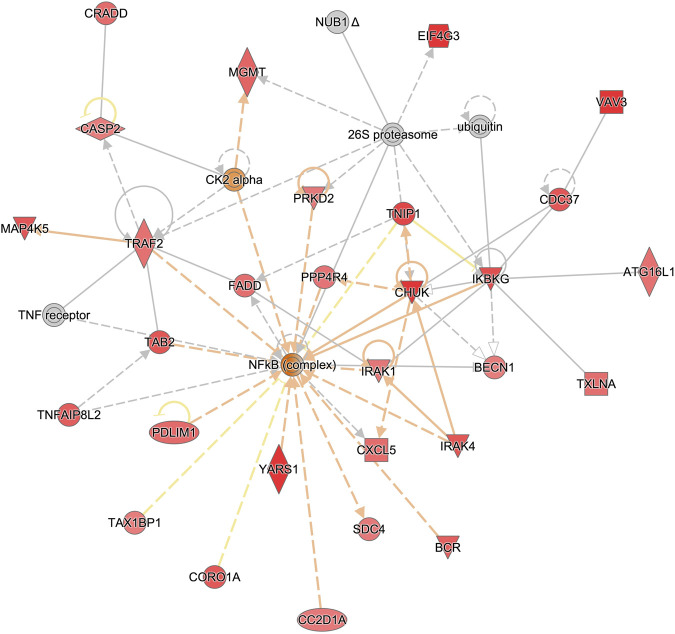
Networks of dysregulated genes related to cardiac necrosis/Cell death, cardiovascular disease, cell signaling.

**FIGURE 3:**
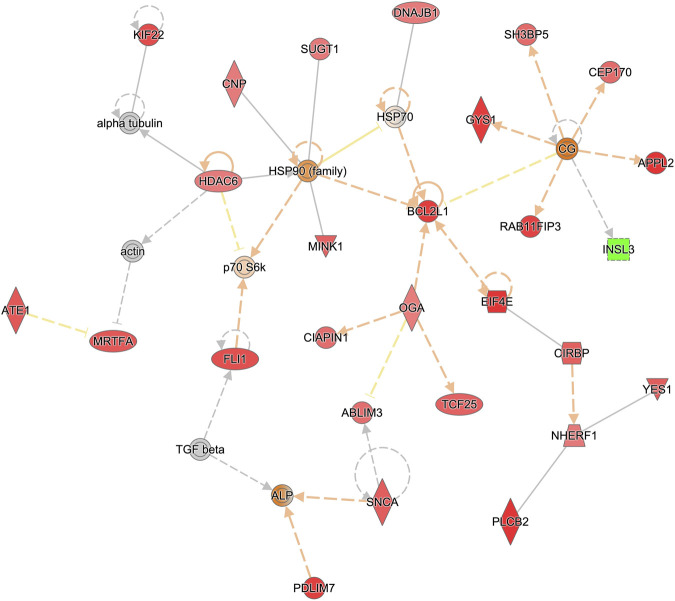
Networks of dysregulated genes related to Cell-To-Cell signaling and interaction, cellular assembly and organization, cellular function and maintenance.

Using IPA, we found two top networks to be dysregulated in cases vs. controls based on network scores, which indicate statistical confidence (Figures 2, 3). The most significant network was related to Cardiac Necrosis/Cell Death, Cardiovascular Disease, Cell Signaling (score = 44), and the second network was related to Cell-To-Cell Signaling and Interaction, Cellular Assembly and Organization, and Cellular Function and Maintenance (score = 37). Additional details from IPA, including the list of all dysregulated proteins and canonical pathways, are provided in Supplementary Tables S1, S2.

## Discussion

This study provides preliminary evidence of a novel panel of proteins related to an MRI marker of advanced brain aging, PAD, among a cohort of young adult, primarily male, cognitively-intact veterans and service members. PAD is itself an imaging-derived metric, computed directly from MRI data via the brain age estimation pipeline, and thus the proteomic associations reported in this manuscript evaluate the relationship between an imaging-derived feature and biomarker signatures. These discovery findings suggest that proteins in peripheral blood can be associated with central activities in the brain as evidenced by MRIs, and that following validation will provide new methods to advance understanding of blood biomarkers of brain related changes. Specifically, in this prelimary study, using an Olink exploratory approach, our data identified unique panels of previously unassociated proteins and protein pathways that are sensitive and specific to advanced brain PAD. Specifically, NFκB and heat-shock proteins were associated with advanced brain aging.

Findings presented here in relatively young and healthy adults support previous findings linking neurological and psyrciatric diseases to PAD, as well as providing additional insights into this MRI based method in younger cohorts (Han et al., 2025; Constantinides et al., 2024; Liew et al., 2023) In this study, the mean absolute error was 4.4 years; however, we note that this value is essentially equivalent to the mean PAD across the sample (−4.4 years), suggesting it reflects characteristics of this particular cohort—who are relatively young, physically active veterans and service members—rather than poor model accuracy *perse*. A negative mean PAD in a healthy, physically fit military sample is consistent with prior literature showing that physical fitness and younger demographic profiles are associated with younger-appearing brains. 38,845,484 Specifically, in the Lothian Birth Cohort, advanced brain PAD was associated with slower walking speed, poorer cognitive function, and increased mortality risk (Cole et al., 2018). Advanced brain PAD is also associated with a greater risk of conversion to AD in older individuals (Elkana and Beheshti, 2025). Other health factors, including type 2 diabetes mellitus and impaired liver and kidney function are also associated with advanced brain age (Franke et al., 2014). Even prenatal factors can influence brain age; a study of the Dutch famine of 1944/1945 showed advanced brain age in individuals exposed to restricted nutrition *inutero* (Franke et al., 2018). Importantly, advanced brain PAD has recently been linked to conversion from normal cognition to MCI a mean of 12-years later (Elkana and Beheshti, 2025).

Further, we showed several proteins, including FGFBP3, CRTAC1, CHUK, DSG3, and INSL3 (base model; Figure 1; Table 3), may be important for differentiating those with advanced PAD from control participants. This finding is supported by preclinical models, FGFBP3 was predominately expressed in the orbitofrontal cortex and shown to be important for neuronal development and mediate anxiety-related behavior (Yamanaka et al., 2011), while inhibition of nuclear factor kappa-B kinase subunit alpha (IKKα), or CHUK, was found to be important for memory consolidation in the hippocampus (Lubin and Sweatt, 2007) and promoting the survival of neurons (Khoshnan et al., 2009). Further, CRTAC1 was shown to be important for synaptic development and function (Beugelink et al., 2024) and was elevated in patients in the week following acute ischemic stroke (Kuwashiro et al., 2021), but decreased in patients with frontotemporal dementia and amyotrophic lateral sclerosis compared to controls (Katzeff et al., 2020). Taken together, our results suggest that proteins important for neuronal function and injury are upregulated in those with accelerated PAD, and require additional studies to explicate the nature of these discovery findings. Although not all expressed in the brain, collectively the aforementioned proteins appear to be important for predicting deviations in brain age.

In our most strongly implicated network, CHUK and IKBKG were included, with both being responsible for activating NF-κB. NF-κB from previous findings, suggested that it may be crucial for aging, as immune response dysregulation leads to chronic systemic inflammation (García et al., 2021). These findings are supported by preliminary data previously showing that chronic inflammation is frequently observed in age-related diseases and related to brain age through reduced synaptic plasticity, memory, and to advanced brain aging (Adler et al., 2007). In preclinical models, administration of agents that downregulate inflammatory signaling pathways, including NF-κB, promote greater synaptic activity within neurons (Aktar et al., 2024). Together with increased TNFα levels, our findings suggest a chronic inflammatory state may be associated with advanced brain PAD, as it is associated with decline in gray matter volume and increase in white matter hyperintensities in older, cognitively intact adults (Lindbergh et al., 2020).

We observed heat-shock protein (HSP) 90 to be a major hub, suggesting over-activity. HSP90 maintains cellular proteostasis and development of neurodegenerative proteinopathies associated with aging-associated functional declines (Wu et al., 2025). Non-human primate models of aging and ADRD show HSP90-specific tracer has strong binding in the brain, with lower activity suggesting greater aging vulnerability (Cools et al., 2025). We also linked HSP70 to PAD, which interacts with HSP90 to ensure cellular proteostasis and regulate cellular aging (Bhattacharya and Picard, 2021), which may relate to the initiation of tau accumulation in the aging brain (Criado-M et al., 2021). BCL2L1 plays a critical role in anti-apoptosis signalling, leading to the survival of injured and aging cells and the accumulation of senescent cells (Hu et al., 2022) and is also linked to aging (Borras et al., 2016). These findings provide insights into the underlying brain changes that may contribute to accelerated brain PAD.

We also observed discovery findings that differences in proteins related to neuronal development and structural plasticity. Specifically, secreted-frizzled-related protein 1 (SFRP-1) distinguished advanced brain PAD. SFRP-1 is a well-established antagonist of Wnt signaling, which plays a crucial role in maintaining the structure and function of the neuronal circuits in the adult hippocampus (Gogolla et al., 2009) and neurodegenerative processes (Fernandez-Berrocal et al., 2025). Dysregulation of SFRP1 has also been identified as a critical link to a greater ADRD risk in the APOE ɛ4 risk allele (Oveisgharan et al., 2024). Furthermore, we observed advanced brain PAD to be associated with upregulation of fasciculation and elongation protein zeta-2 (FEZ2), a protein essential to brain health, including microtubule stabilization in axonal growth and synaptic organization (Truvé et al., 2020). Thus, these findings suggest that biomarker pathways related to neuronal proliferation may contribute to discordant brain PAD, and warrant additional studies to understand these likely complex interactions.

The networks we found related to advanced brain aging further implicate pathological processes related to AD. α-synuclein (SNCA) proteins, found in Figure 3 network, may be a potential novel target to investigate the role of *GBA* and SNCA in cognitive decline. *GBA* is known as a major risk gene for PD and dementia with Lewy bodies. Gba mutations trigger SNCA aggregation leading to Lewy body formation (Vidyadhara et al., 2025) and development of cognitive decline. Key deregulated proteins in the network, including SNCA, were linked to neurodegenerative pathways, suggesting molecular connections between advanced brain PAD and diseases like AD and PD. These protein networks were related to possible advanced aging pathologies, suggesting the need for future studies examining the role in advanced brain PAD.

The current study has several notable limitations, including a cross-sectional design and a modest sample size, which limits interpretations of findings. Further, this is a discovery-based study where protein panels and pathways were identified, but there was no in-laboratory validation included, nor is there a clinical validation within another cohort. These limitations suggest additional longintundial studies that generate quantitative proteomics to improve confidence on the proteins implicated in this discovery cohort. Also, Olink Explorer generates data that is relative in level, not quantitative, which limits model building as well as understanding biomarker interactions fully. In future studies we will include additional imaging features (e.g., regional volumes, cortical thickness, white matter microstructure) in relation to the proteomic data, but it is not within the scope of the present study, which focuses on PAD as a global summary index of brain aging. Our study participants were mostly male and restricted to service members and veterans. Further, brain PAD is newer concept, which does not have the same consistent methods as other MRI metrics, yet it has beenis a widely utilized open-source tool that has demonstrated high accuracy and exceptional test-retest reliability (ICC 0.94–0.98) relative to other brain age models, provided that motion artifacts are adequately addressed. 28,765,056 Further, it has demonstrated that different brain age models tend to produce highly correlated PAD estimates, suggesting that downstream associations—such as the proteomic relationships reported here—are likely to reflect consistent biological signal rather than method-specific artifact. 28,765,056 BrainageR has been extensively validated and benchmarked against other tools in the studies cited above, and its open-source availability supports reproducibility. 35,312,210 (3) BrainageR has demonstrated robustness across scanner field strengths (1.5T and 3T) and consistency in longitudinal analyses (Franke and Gaser, 2019), supporting generalizability across imaging protocols31474922. Future research should examine other brain age algorithms to gain additional detail into what brain changes are driving differences in brain age calculations, especially within longitudinal studies that link these biomarkers to clinical implications over time. Nonetheless, this exciting line of research shows promise for blood protein assay methods of detecting accelerated brain age prior to pathological changes and identify patients who may benefit from early interventions.

## Data Availability

The affinity proteomics data have been deposited to the PRIDE repository with the dataset identifier PAD000050.

## References

[B1] Adler A. S. Sinha S. Kawahara T. L. Zhang J. Y. Segal E. Chang H. Y. (2007). Motif module map reveals enforcement of aging by continual NF-kappaB activity. Genes Dev. 21 (24), 3244–3257. 10.1101/gad.1588507 18055696 PMC2113026

[B2] Aktar S. Kato A. Toda K. Takahashi S. Maeda-Yamamoto M. Ferdousi F. (2024). Transcriptomic evidence of black soybean ethanolic extract and 2-aminobutyric acid in suppressing neuroinflammation and enhancing synaptic transmission. Biomed. Pharmacother. 181, 117633. 10.1016/j.biopha.2024.117633 39488055

[B3] Anjum T. Seyfizadeh A. Ding H. Mahmood T. Pryss R. Volkmann J. (2025). Directed brain connectivity biomarkers of healthy aging and Parkinson's disease staging. Front. Aging Neurosci. 17, 1698600. 10.3389/fnagi.2025.1698600 41229609 PMC12602468

[B4] Beugelink J. W. Hóf H. Janssen B. J. C. (2024). CRTAC1 has a compact β-propeller-TTR core stabilized by potassium ions. J. Mol. Biol. 436 (18), 168712. 10.1016/j.jmb.2024.168712 39029889

[B5] Beydoun M. A. Beydoun H. A. Fanelli-Kuczmarski M. T. Hu Y. H. Shaked D. Weiss J. (2025). Uncovering mediational pathways behind racial and socioeconomic disparities in brain volumes: insights from the UK Biobank study. Geroscience 47 (2), 1837–1858. 10.1007/s11357-024-01371-1 39388067 PMC11979012

[B6] Bhattacharya K. Picard D. (2021). The Hsp70-Hsp90 go-between Hop/Stip1/Sti1 is a proteostatic switch and may be a drug target in cancer and neurodegeneration. Cell Mol. Life Sci. 78 (23), 7257–7273. 10.1007/s00018-021-03962-z 34677645 PMC8629791

[B7] Borras C. Abdelaziz K. M. Gambini J. Serna E. Inglés M. de la Fuente M. (2016). Human exceptional longevity: transcriptome from centenarians is distinct from septuagenarians and reveals a role of Bcl-xL in successful aging. Aging (Albany NY) 8 (12), 3185–3208. 10.18632/aging.101078 27794564 PMC5270663

[B8] Cai M. He C. Li H. Yang R. Rong S. Gao Z. (2025). Advanced brain aging, selective vulnerability in gray matter, and cognition in Parkinson's disease. J. Gerontol. A Biol. Sci. Med. Sci. 80 (9), glaf124. 10.1093/gerona/glaf124 40493890 PMC12398390

[B9] Casanova R. Walker K. A. Justice J. N. Anderson A. Duggan M. R. Cordon J. (2024). Associations of plasma proteomics and age-related outcomes with brain age in a diverse cohort. Geroscience 46 (4), 3861–3873. 10.1007/s11357-024-01112-4 38438772 PMC11226584

[B10] Cifu D. X. (2022/04/16 2022). Clinical research findings from the long-term impact of military-relevant brain injury consortium-Chronic Effects of Neurotrauma Consortium (LIMBIC-CENC) 2013-2021. Brain Inj. 36 (5), 587–597. 10.1080/02699052.2022.2033843 35080997

[B11] Cole J. H. Leech R. Sharp D. J. (2015). Prediction of brain age suggests accelerated atrophy after traumatic brain injury. Ann. Neurol. 77 (4), 571–581. 10.1002/ana.24367 25623048 PMC4403966

[B12] Cole J. H. Ritchie S. J. Bastin M. E. Valdés Hernández M. C. Muñoz Maniega S. Royle N. (2018). Brain age predicts mortality. Mol. Psychiatry 23 (5), 1385–1392. 10.1038/mp.2017.62 28439103 PMC5984097

[B13] Constantinides C. Caramaschi D. Zammit S. Freeman T. P. Walton E. (2024). Exploring Associations between Psychotic Experiences and Structural Brain age: A Population-based study in Late Adolescence. medRxiv. 10.1101/2024.10.07.24314890

[B14] Cools R. Vermeulen K. Vonck E. Baekelandt V. Varlow C. Narykina V. (2025). *In vivo* visualization and quantification of brain heat shock protein 90 with [(11)C]HSP990 in healthy aging and neurodegeneration. J. Nucl. Med. 66 (6), 940–947. 10.2967/jnumed.124.268961 40306968 PMC12175989

[B15] Coors A. Boenniger M. M. Santos M. L. S. Lohner V. Koch A. Ettinger U. (2025). Associations of plasma neurofilament light levels with brain microstructure and macrostructure and cognition in the community-based rhineland study. Neurology 104 (6), e210278. 10.1212/wnl.0000000000210278 39977717

[B16] Criado-Marrero M. Gebru N. T. Blazier D. M. Gould L. A. Baker J. D. Beaulieu-Abdelahad D. (2021). Hsp90 co-chaperones, FKBP52 and Aha1, promote tau pathogenesis in aged wild-type mice. Acta Neuropathol. Commun. 9 (1), 65. 10.1186/s40478-021-01159-w 33832539 PMC8033733

[B18] de Souza N. L. Lindsey H. M. Dorman K. Dennis E. L. Kennedy E. Menefee D. S. (2024). Neuropsychological profiles of deployment-related mild traumatic brain injury: a LIMBIC-CENC study. Neurology 102 (12), e209417. 10.1212/wnl.0000000000209417 38833650 PMC11226312

[B19] Elkana O. Beheshti I. (2025). Brain age as an accurate biomarker of preclinical cognitive decline: evidence from a 12-year longitudinal study. J. Neurol. 272 (10), 672. 10.1007/s00415-025-13414-4 41039123

[B20] Fernandez-Berrocal M. S. Reis A. Rolseth V. Suganthan R. Kuśnierczyk A. França A. (2025). NEIL3 influences adult neurogenesis and behavioral pattern separation *via* WNT signaling. Cell Mol. Life Sci. 82 (1), 101. 10.1007/s00018-025-05629-5 40035863 PMC11880487

[B21] Franke K. Ristow M. Gaser C., and Alzheimer's Disease Neuroimaging Initiative (2014). Gender-specific impact of personal health parameters on individual brain aging in cognitively unimpaired elderly subjects. Front. Aging Neurosci. 6, 94. 10.3389/fnagi.2014.00094 24904408 PMC4033192

[B22] Franke K. Gaser C. Roseboom T. J. Schwab M. de Rooij S. R. (2018). Premature brain aging in humans exposed to maternal nutrient restriction during early gestation. Neuroimage. 173, 460–471. 10.1016/j.neuroimage.2017.10.047 29074280

[B23] García-García V. A. Alameda J. P. Page A. Casanova M. L. (2021). Role of NF-κB in ageing and age-related diseases: lessons from genetically modified mouse models. Cells 10 (8), 1906. 10.3390/cells10081906 34440675 PMC8394846

[B24] Gogolla N. Galimberti I. Deguchi Y. Caroni P. (2009). Wnt signaling mediates experience-related regulation of synapse numbers and mossy fiber connectivities in the adult hippocampus. Neuron 62 (4), 510–525. 10.1016/j.neuron.2009.04.022 19477153

[B25] Han L. K. M. Toenders Y. J. Shen X. Milaneschi Y. Whalley H. C. Sämann P. G. (2025). Lifestyle, early-life, and Genetic Health Risk Factors Underlying the Brain Age Gap: A Mega-Analysis Across 3,934 Individuals from the ENIGMA MDD Consortium. 10.1101/2025.05.09.653064

[B26] Haugg F. Lee G. He J. Johnson J. Zapaishchykova A. Bitterman D. S. (2025). Imaging biomarkers of ageing: a review of artificial intelligence-based approaches for age estimation. Lancet Healthy Longev. 6 (7), 100728. 10.1016/j.lanhl.2025.100728 40690918

[B28] Hu L. Li H. Zi M. Li W. Liu J. Yang Y. (2022). Why senescent cells are resistant to apoptosis: an insight for senolytic development. Front. Cell Dev. Biol. 10, 822816. 10.3389/fcell.2022.822816 35252191 PMC8890612

[B30] Katzeff J. S. Bright F. Lo K. Kril J. J. Connolly A. Crossett B. (2020). Altered serum protein levels in frontotemporal dementia and amyotrophic lateral sclerosis indicate calcium and immunity dysregulation. Sci. Rep. 10 (1), 13741. 10.1038/s41598-020-70687-7 32792518 PMC7426269

[B31] Khoshnan A. Ko J. Tescu S. Brundin P. Patterson P. H. (2009). IKKalpha and IKKbeta regulation of DNA damage-induced cleavage of huntingtin. PLoS One 4 (6), e5768. 10.1371/journal.pone.0005768 19488402 PMC2685016

[B32] Kuwashiro T. Tanabe K. Hayashi C. Mizoguchi T. Mori K. Jinnouchi J. (2021). Oxidized albumin and cartilage acidic Protein-1 as blood biomarkers to predict ischemic stroke outcomes. Front. Neurol. 12, 686555. 10.3389/fneur.2021.686555 34917008 PMC8670551

[B33] Liew S. L. Schweighofer N. Cole J. H. Zavaliangos-Petropulu A. Tavenner B. P. Han L. K. M. (2023). Association of brain age, lesion volume, and functional outcome in patients with stroke. Neurology 100 (20), e2103–e2113. 10.1212/wnl.0000000000207219 37015818 PMC10186236

[B34] Lindbergh C. A. Casaletto K. B. Staffaroni A. M. Elahi F. Walters S. M. You M. (2020). Systemic tumor necrosis factor-alpha trajectories relate to brain health in typically aging older adults. J. Gerontol. A Biol. Sci. Med. Sci. 75 (8), 1558–1565. 10.1093/gerona/glz209 31549145 PMC7457183

[B35] Lubin F. D. Sweatt J. D. (2007). The IkappaB kinase regulates chromatin structure during reconsolidation of conditioned fear memories. Neuron 55 (6), 942–957. 10.1016/j.neuron.2007.07.039 17880897 PMC2587178

[B36] Namsrai T. Northey J. M. Ambikairajah A. Ahmed O. Alateeq K. Espinoza Oyarce D. A. (2025). Sleep characteristics and brain structure: a systematic review with meta-analysis. Sleep. Med. 129, 316–329. 10.1016/j.sleep.2025.02.028 40086297

[B37] Oveisgharan S. Yu L. de Paiva Lopes K. Tasaki S. Wang Y. Menon V. (2024). Proteins linking APOE ɛ4 with alzheimer's disease. Alzheimers Dement. 20 (7), 4499–4511. 10.1002/alz.13867 38856164 PMC11247662

[B38] Pahlke S. Kahale L. A. Mahinrad S. Sousa-Pinto B. Vieira R. J. McAteer M. B. (2025). Blood-based biomarkers for detecting alzheimer's disease pathology in cognitively impaired individuals within specialized care settings: a systematic review and meta-analysis. Alzheimers Dement. 21 (11), e70828. 10.1002/alz.70828 41193403 PMC12590577

[B39] Papouli A. Cole J. H. (2025). Brain age prediction from MRI scans in neurodegenerative diseases. Curr. Opin. Neurol. 38 (4), 316–321. 10.1097/wco.0000000000001383 40396549 PMC12237103

[B40] Pilli R. Goel T. Murugan R. (2025). Unveiling alzheimer's disease through brain age estimation using multi-kernel regression network and magnetic resonance imaging. Comput. Methods Programs Biomed. 261, 108617. 10.1016/j.cmpb.2025.108617 39908635

[B43] Sim M. A. Rowsthorn E. O'Brien W. T. Sun M. Cribb L. Franks K. (2025). Plasma phosphorylated tau-217 correlates with brain atrophy, cognition, and cerebrospinal fluid biomarkers in a cognitively healthy community cohort. Brain Commun. 7 (5), fcaf383. 10.1093/braincomms/fcaf383 41140811 PMC12550561

[B44] Truvé K. Parris T. Z. Vizlin-Hodzic D. Salmela S. Berger E. Ågren H. (2020). Identification of candidate genetic variants and altered protein expression in neural stem and mature neural cells support altered microtubule function to be an essential component in bipolar disorder. Transl. Psychiatry 10 (1), 390. 10.1038/s41398-020-01056-1 33168801 PMC7652854

[B46] Vidyadhara D. J. Bäckström D. Chakraborty R. Ruan J. Park J. M. Mistry P. K. (2025). Synaptic vesicle endocytosis deficits underlie cognitive dysfunction in mouse models of GBA-Linked parkinson's disease and dementia with lewy bodies. Nat. Commun. 16 (1), 8484. 10.1038/s41467-025-63444-9 41006254 PMC12475166

[B47] Vogt D. Smith B. N. King L. A. King D. W. Knight J. Vasterling J. J. (2013). Deployment risk and resilience inventory-2 (DRRI-2): an updated tool for assessing psychosocial risk and resilience factors among service members and veterans. J. Trauma Stress 26 (6), 710–717. 10.1002/jts.21868 24490250

[B48] Walker W. C. Carne W. Franke L. M. Nolen T. Dikmen S. D. Cifu D. X. (2016). The chronic effects of neurotrauma consortium (CENC) multi-centre observational study: description of study and characteristics of early participants. Brain Inj. 30 (12), 1469–1480. 10.1080/02699052.2016.1219061 27834538

[B49] Wu J. Yarmey V. R. Yang O. J. Soderblom E. J. San-Miguel A. Yan D. (2025). Heat shock proteins function as signaling molecules to mediate neuron-glia communication in *C. elegans* during aging. Nat. Neurosci. 28 (8), 1635–1648. 10.1038/s41593-025-01989-0 40533573

[B50] Yamanaka Y. Kitano A. Takao K. Prasansuklab A. Mushiroda T. Yamazaki K. (2011). Inactivation of fibroblast growth factor binding protein 3 causes anxiety-related behaviors. Mol. Cell Neurosci. 46 (1), 200–212. 10.1016/j.mcn.2010.09.003 20851768

