# Supplementary material for: A discovery protein panel for brain predicted age discordance using MRI in neurologically healthy individuals
Source: Front Cell Dev Biol. 2026 Jul 9;14:1833866. doi: 10.3389/fcell.2026.1833866 (PMC13392082; doi:10.3389/fcell.2026.1833866)
Supplement: Supplementary file 1 [file DataSheet1.pdf]

**Supplementary Table 1. Dysregulated Proteins**

Significantly dysregulated proteins included in this analysis. Proteins with adjusted p-value <0.05 and Log2 fold change greater than 1 were considered significant.

| Proteins | log2Fold Change | Log2 Average Control Group | Log2 Average Case Group | p.value | Adjusted_pval |
|---|---|---|---|---|---|
| CHUK | 1.80E+00 | -6.57E-01 | 1.15E+00 | 5.71E-08 | 9.61E-05 |
| MPIG6B | 1.41E+00 | -4.83E-01 | 9.29E-01 | 7.08E-08 | 9.61E-05 |
| ASAP3 | 1.07E+00 | -2.85E-01 | 7.81E-01 | 1.62E-07 | 1.76E-04 |
| INSL3 | -2.27E+00 | -6.37E-01 | -2.91E+00 | 3.21E-07 | 2.36E-04 |
| FEZ2 | 1.17E+00 | -5.26E-01 | 6.46E-01 | 7.04E-07 | 2.55E-04 |
| PMM2 | 1.27E+00 | -3.45E-01 | 9.23E-01 | 7.04E-07 | 2.55E-04 |
| EGF | 1.70E+00 | -5.37E-01 | 1.16E+00 | 9.46E-07 | 2.57E-04 |
| IKBKG | 1.38E+00 | -4.94E-01 | 8.90E-01 | 9.77E-07 | 2.57E-04 |
| NEDD1 | 1.27E+00 | -3.82E-01 | 8.91E-01 | 8.44E-07 | 2.57E-04 |
| RNF41 | 1.04E+00 | -2.51E-01 | 7.84E-01 | 9.94E-07 | 2.57E-04 |
| PZP | 1.07E+00 | -1.12E-01 | 9.56E-01 | 1.11E-06 | 2.75E-04 |
| PRKD2 | 1.04E+00 | -3.66E-01 | 6.75E-01 | 1.17E-06 | 2.76E-04 |
| ARHGEF12 | 1.61E+00 | -6.82E-01 | 9.27E-01 | 1.37E-06 | 2.81E-04 |
| BACH1 | 1.12E+00 | -1.41E-01 | 9.84E-01 | 1.61E-06 | 2.81E-04 |
| DUSP19 | 1.08E+00 | -2.21E-01 | 8.59E-01 | 1.59E-06 | 2.81E-04 |
| GEMIN2 | 1.36E+00 | -4.07E-01 | 9.55E-01 | 1.29E-06 | 2.81E-04 |
| MTHFSD | 1.78E+00 | -6.17E-01 | 1.17E+00 | 1.83E-06 | 2.81E-04 |
| SULT1A1 | 1.47E+00 | -4.07E-01 | 1.06E+00 | 1.40E-06 | 2.81E-04 |
| THTPA | 1.20E+00 | -3.19E-01 | 8.85E-01 | 1.70E-06 | 2.81E-04 |
| TSC1 | 1.26E+00 | -6.68E-01 | 5.88E-01 | 1.66E-06 | 2.81E-04 |
| GYS1 | 1.530405 | -0.37652 | 1.153884 | 2.21E-06 | 0.00031 |
| SPINT3 | -2.00463 | -0.22928 | -2.23391 | 2.29E-06 | 0.00031 |
| NRGN | 1.515929 | -0.40365 | 1.112279 | 2.55E-06 | 0.000338 |
| CMIP | 1.579092 | -0.51283 | 1.066258 | 2.80E-06 | 0.000354 |
| CD69 | 1.3612 | -0.33177 | 1.029427 | 2.94E-06 | 0.00036 |
| SDC4 | 1.034092 | -0.16516 | 0.868929 | 2.98E-06 | 0.00036 |
| BRAP | 1.407962 | -0.49582 | 0.912147 | 3.59E-06 | 0.000372 |
| FYCO1 | 1.726556 | -0.65757 | 1.068986 | 3.97E-06 | 0.000372 |
| INPPL1 | 1.474903 | -0.52197 | 0.95293 | 3.94E-06 | 0.000372 |
| MTSS1 | 1.322642 | -0.51096 | 0.811683 | 3.38E-06 | 0.000372 |
| PER3 | 1.255001 | -0.46582 | 0.789182 | 3.88E-06 | 0.000372 |
| SDCCAG8 | 1.673567 | -0.70009 | 0.973474 | 3.59E-06 | 0.000372 |
| TBL1X | 1.366399 | -0.51507 | 0.851332 | 3.94E-06 | 0.000372 |
| UBOX5 | 1.054944 | -0.2448 | 0.810144 | 3.94E-06 | 0.000372 |
| DGKA | 1.059071 | -0.29983 | 0.759244 | 4.13E-06 | 0.000373 |
| ANKS1A | 1.043505 | -0.11065 | 0.932856 | 4.46E-06 | 0.000379 |
| CKAP5 | 1.372601 | -0.48281 | 0.889789 | 4.36E-06 | 0.000379 |

| Proteins | log2Fold Change | Log2 Average Control Group | Log2 Average Case Group | p.value | Adjusted_pval |
|---|---|---|---|---|---|
| AZI2 | 1.29768 | -0.30197 | 0.995711 | 4.60E-06 | 0.000384 |
| MZT2B | 1.176987 | -0.30484 | 0.872147 | 4.96E-06 | 0.000402 |
| ARHGAP10 | 1.453462 | -0.69012 | 0.763345 | 6.33E-06 | 0.000411 |
| ARHGAP45 | 1.437719 | -0.59135 | 0.84637 | 6.33E-06 | 0.000411 |
| ARL2BP | 1.080283 | -0.39415 | 0.686131 | 6.43E-06 | 0.000411 |
| CD40LG | 1.073193 | -0.25354 | 0.819658 | 6.05E-06 | 0.000411 |
| CNST | 1.148605 | -0.19338 | 0.955221 | 5.69E-06 | 0.000411 |
| EIF2AK3 | 1.399252 | -0.2684 | 1.130852 | 5.52E-06 | 0.000411 |
| EIF2B4 | 1.204669 | -0.42853 | 0.776134 | 5.78E-06 | 0.000411 |
| GMFG | 1.402058 | -0.53419 | 0.867871 | 5.96E-06 | 0.000411 |
| GOPC | 1.506865 | -0.59676 | 0.910104 | 6.33E-06 | 0.000411 |
| SAV1 | 1.190775 | -0.34849 | 0.842286 | 6.43E-06 | 0.000411 |
| TMSB10 | 1.023762 | -0.28418 | 0.739585 | 6.23E-06 | 0.000411 |
| TSC22D3 | 1.499911 | -0.64034 | 0.859572 | 6.43E-06 | 0.000411 |
| VASH1 | 1.1441 | -0.03834 | 1.10576 | 5.69E-06 | 0.000411 |
| DDHD2 | 1.04427 | -0.34469 | 0.69958 | 6.72E-06 | 0.000425 |
| TRAF2 | 1.0967 | -0.35349 | 0.743208 | 6.83E-06 | 0.000426 |
| CETN2 | 1.195819 | -0.30143 | 0.894391 | 7.14E-06 | 0.000431 |
| STX17 | 1.254949 | -0.15256 | 1.102393 | 7.25E-06 | 0.000433 |
| DCTN6 | 1.137011 | -0.11758 | 1.019433 | 7.93E-06 | 0.000444 |
| DDX19B | 1.287347 | -0.39367 | 0.89368 | 7.93E-06 | 0.000444 |
| INPP4B | 1.799037 | -0.81683 | 0.982211 | 7.93E-06 | 0.000444 |
| TREML1 | 1.292456 | -0.227 | 1.065453 | 7.70E-06 | 0.000444 |
| NFATC1 | 1.228464 | -0.36286 | 0.865608 | 8.30E-06 | 0.000455 |
| MTSS2 | 1.227845 | -0.15319 | 1.074654 | 8.42E-06 | 0.000455 |
| PSTPIP2 | 1.585695 | -0.89744 | 0.688255 | 8.55E-06 | 0.000455 |
| UNC45A | 1.273198 | -0.38939 | 0.883811 | 8.94E-06 | 0.000462 |
| ZNRD2 | 1.326523 | -0.20121 | 1.125311 | 8.94E-06 | 0.000462 |
| AAGAB | 1.209379 | -0.37348 | 0.835901 | 9.48E-06 | 0.000464 |
| EEF1D | 1.405889 | -0.33872 | 1.067167 | 9.41E-06 | 0.000464 |
| FOXO1 | 1.158771 | -0.34341 | 0.815357 | 9.34E-06 | 0.000464 |
| GOLGA3 | 1.152413 | -0.24514 | 0.907272 | 9.48E-06 | 0.000464 |
| RRM2B | 1.005538 | -0.3346 | 0.670936 | 9.07E-06 | 0.000464 |
| EML4 | 1.377056 | -0.46235 | 0.914711 | 9.77E-06 | 0.00047 |
| LRBA | 1.445516 | -0.61452 | 0.830993 | 1.02E-05 | 0.000474 |
| MAVS | 1.401945 | -0.48362 | 0.918324 | 1.02E-05 | 0.000474 |
| ZC3H12A | 1.011254 | -0.23475 | 0.776502 | 1.01E-05 | 0.000474 |
| ARHGAP35 | 1.219376 | -0.24815 | 0.971231 | 1.08E-05 | 0.000486 |
| CRACR2A | 1.585069 | -0.45367 | 1.131397 | 1.10E-05 | 0.000486 |
| EVI5 | 1.025582 | -0.20054 | 0.825045 | 1.08E-05 | 0.000486 |

| Proteins | log2Fold Change | Log2 Average Control Group | Log2 Average Case Group | p.value | Adjusted_pval |
|---|---|---|---|---|---|
| MYH9 | 1.348909 | -0.21662 | 1.132286 | 1.08E-05 | 0.000486 |
| CASP3 | 1.475195 | -0.54973 | 0.925469 | 1.18E-05 | 0.000487 |
| HPCAL1 | 1.005608 | -0.38604 | 0.619567 | 1.13E-05 | 0.000487 |
| IRAK1 | 1.05596 | -0.1958 | 0.86016 | 1.15E-05 | 0.000487 |
| TRIM25 | 1.414808 | -0.51568 | 0.899124 | 1.17E-05 | 0.000487 |
| BANK1 | 1.333239 | -0.50056 | 0.832679 | 1.22E-05 | 0.000494 |
| EVI5L | 1.732791 | -0.71326 | 1.019536 | 1.22E-05 | 0.000494 |
| TRAFD1 | 1.454766 | -0.50983 | 0.944932 | 1.24E-05 | 0.000494 |
| RABEP1 | 1.218615 | -0.20377 | 1.014843 | 1.29E-05 | 0.000502 |
| RAB3IP | 1.562614 | -0.50416 | 1.058456 | 1.32E-05 | 0.000509 |
| CALCOCO1 | 1.585921 | -0.73836 | 0.847565 | 1.54E-05 | 0.00051 |
| CAMSAP1 | 1.614862 | -0.63789 | 0.976974 | 1.53E-05 | 0.00051 |
| ESYT1 | 1.502525 | -0.3879 | 1.114628 | 1.54E-05 | 0.00051 |
| KAZN | 1.151165 | -0.4571 | 0.694062 | 1.52E-05 | 0.00051 |
| MAP2K6 | 1.175032 | -0.38329 | 0.79174 | 1.54E-05 | 0.00051 |
| OGA | 1.011564 | -0.1849 | 0.826661 | 1.54E-05 | 0.00051 |
| OPHN1 | 1.436418 | -0.63228 | 0.804133 | 1.54E-05 | 0.00051 |
| PACS2 | 1.169035 | -0.32863 | 0.840408 | 1.35E-05 | 0.00051 |
| PCBP2 | 1.155805 | -0.37091 | 0.784895 | 1.52E-05 | 0.00051 |
| PLPBP | 1.142615 | -0.26598 | 0.876637 | 1.39E-05 | 0.00051 |
| RELCH | 1.526393 | -0.56004 | 0.966349 | 1.47E-05 | 0.00051 |
| RILPL1 | 1.04624 | -0.17663 | 0.869615 | 1.41E-05 | 0.00051 |
| SERPINB1 | 1.136102 | -0.20237 | 0.933727 | 1.52E-05 | 0.00051 |
| USP25 | 1.205875 | -0.32306 | 0.882817 | 1.52E-05 | 0.00051 |
| MRTFA | 1.311715 | -0.36944 | 0.942276 | 1.56E-05 | 0.000511 |
| SCAMP2 | 1.088763 | -0.19422 | 0.894546 | 1.56E-05 | 0.000511 |
| CASP2 | 1.017127 | -0.19529 | 0.82184 | 1.70E-05 | 0.000523 |
| CPPED1 | 1.015411 | -0.11976 | 0.895652 | 1.66E-05 | 0.000523 |
| DBNL | 1.548851 | -0.56343 | 0.985419 | 1.70E-05 | 0.000523 |
| MICAL1 | 1.153606 | -0.29156 | 0.862047 | 1.68E-05 | 0.000523 |
| NAA10 | 1.174901 | -0.35822 | 0.81668 | 1.68E-05 | 0.000523 |
| TNIP1 | 1.462134 | -0.45257 | 1.009561 | 1.63E-05 | 0.000523 |
| TRAK1 | 1.080752 | -0.17141 | 0.909341 | 1.64E-05 | 0.000523 |
| ANKMY2 | 1.350487 | -0.46727 | 0.883218 | 1.81E-05 | 0.000527 |
| ARFIP1 | 1.156634 | -0.34882 | 0.807815 | 1.81E-05 | 0.000527 |
| CNN2 | 1.252882 | -0.53716 | 0.715726 | 1.73E-05 | 0.000527 |
| DAP | 1.594029 | -0.26683 | 1.3272 | 1.78E-05 | 0.000527 |
| GIT1 | 1.469221 | -0.66569 | 0.803535 | 1.81E-05 | 0.000527 |
| RABEP2 | 1.357027 | -0.39602 | 0.961002 | 1.81E-05 | 0.000527 |
| TJAP1 | 1.199133 | -0.20931 | 0.989819 | 1.74E-05 | 0.000527 |

| Proteins | log2Fold Change | Log2 Average Control Group | Log2 Average Case Group | p.value | Adjusted_pval |
|---|---|---|---|---|---|
| MARS1 | 1.244461 | -0.20391 | 1.040549 | 1.83E-05 | 0.000531 |
| PRKAR1A | 1.250783 | -0.31667 | 0.934114 | 1.86E-05 | 0.000531 |
| KIF22 | 1.409236 | -0.41042 | 0.998818 | 1.87E-05 | 0.000532 |
| DCTD | 1.46317 | -0.57597 | 0.887199 | 1.88E-05 | 0.000533 |
| GTPBP2 | 1.696899 | -0.59945 | 1.097448 | 1.90E-05 | 0.000534 |
| MAP4K5 | 1.321032 | -0.39845 | 0.922586 | 2.00E-05 | 0.00055 |
| PLA2G4A | 1.262387 | -0.35853 | 0.90386 | 2.00E-05 | 0.00055 |
| AXIN1 | 1.378135 | -0.54131 | 0.836827 | 2.08E-05 | 0.000558 |
| BCR | 1.244135 | -0.25119 | 0.992946 | 2.08E-05 | 0.000558 |
| CHMP1A | 1.190665 | -0.30799 | 0.882672 | 2.05E-05 | 0.000558 |
| PLEKHO1 | 1.386148 | -0.54082 | 0.845328 | 2.08E-05 | 0.000558 |
| STAT5B | 1.865838 | -0.44427 | 1.421573 | 2.05E-05 | 0.000558 |
| TTF2 | 1.134875 | -0.39946 | 0.735411 | 2.08E-05 | 0.000558 |
| AK2 | 1.773689 | -0.90255 | 0.871143 | 2.11E-05 | 0.00056 |
| EIF2AK2 | 1.646576 | -0.5626 | 1.083973 | 2.14E-05 | 0.000566 |
| PTRHD1 | 1.252208 | -0.47603 | 0.776182 | 2.24E-05 | 0.000571 |
| SPRED2 | 1.050357 | -0.35259 | 0.69777 | 2.24E-05 | 0.000571 |
| TNFAIP8L2 | 1.263307 | -0.36629 | 0.897013 | 2.27E-05 | 0.000571 |
| FADD | 1.116479 | -0.21113 | 0.905346 | 2.30E-05 | 0.000577 |
| CIAPIN1 | 1.14228 | -0.26544 | 0.876835 | 2.40E-05 | 0.000593 |
| WASF1 | 1.38094 | -0.40397 | 0.976975 | 2.51E-05 | 0.000603 |
| TRIM41 | 1.022098 | -0.29572 | 0.726377 | 2.54E-05 | 0.000609 |
| YES1 | 1.262348 | -0.31971 | 0.942637 | 2.58E-05 | 0.000615 |
| MITD1 | 1.411855 | -0.63555 | 0.776309 | 2.77E-05 | 0.000646 |
| EHD4 | 1.028287 | -0.42894 | 0.599351 | 2.81E-05 | 0.000647 |
| STK39 | 1.295841 | -0.32349 | 0.972349 | 2.81E-05 | 0.000647 |
| UBR2 | 1.568026 | -0.7462 | 0.821828 | 2.85E-05 | 0.00065 |
| AFAP1L2 | 1.124906 | -0.25906 | 0.865844 | 2.89E-05 | 0.000654 |
| EIF4G3 | 1.723187 | -0.57538 | 1.147806 | 2.93E-05 | 0.000655 |
| NUB1 | 1.023311 | -0.16541 | 0.857897 | 2.93E-05 | 0.000655 |
| USP8 | 1.243473 | -0.25987 | 0.983603 | 2.97E-05 | 0.000659 |
| ASCC1 | 1.381195 | -0.49184 | 0.889352 | 3.14E-05 | 0.000675 |
| ATG16L1 | 1.103695 | -0.27695 | 0.826742 | 3.14E-05 | 0.000675 |
| CLEC1B | 1.052581 | -0.07196 | 0.980623 | 3.14E-05 | 0.000675 |
| FOXO3 | 1.017522 | -0.33656 | 0.680957 | 3.14E-05 | 0.000675 |
| IRAK4 | 1.327807 | -0.30599 | 1.021815 | 3.14E-05 | 0.000675 |
| PTPN1 | 1.241495 | -0.31968 | 0.921816 | 3.10E-05 | 0.000675 |
| CRYZL1 | 1.263239 | -0.48595 | 0.777291 | 3.23E-05 | 0.000683 |
| CTIF | 1.476191 | -0.46706 | 1.009132 | 3.23E-05 | 0.000683 |
| GGACT | 1.219382 | -0.37547 | 0.843914 | 3.28E-05 | 0.000688 |

| Proteins | log2Fold Change | Log2 Average Control Group | Log2 Average Case Group | p.value | Adjusted_pval |
|---|---|---|---|---|---|
| ANXA4 | 1.095139 | -0.16737 | 0.927772 | 3.32E-05 | 0.000692 |
| EHBP1 | 1.052452 | -0.18569 | 0.866757 | 3.32E-05 | 0.000692 |
| KATNB1 | 1.202534 | -0.29899 | 0.903545 | 3.47E-05 | 0.000713 |
| AKT2 | 1.827955 | -0.25385 | 1.574106 | 3.57E-05 | 0.000717 |
| CEP104 | 1.328636 | -0.39852 | 0.930113 | 3.57E-05 | 0.000717 |
| JPT2 | 1.147549 | -0.29952 | 0.848026 | 3.57E-05 | 0.000717 |
| TAX1BP1 | 1.008914 | -0.15834 | 0.850574 | 3.57E-05 | 0.000717 |
| BAX | 1.044078 | -0.00941 | 1.034666 | 3.77E-05 | 0.000723 |
| ENSA | 1.408358 | -0.896 | 0.512359 | 3.77E-05 | 0.000723 |
| LRCH1 | 1.139628 | -0.27431 | 0.865315 | 3.67E-05 | 0.000723 |
| MANF | 1.857216 | -0.52175 | 1.33547 | 3.72E-05 | 0.000723 |
| MAPK8IP3 | 1.616799 | -1.04015 | 0.576645 | 3.62E-05 | 0.000723 |
| PRTFDC1 | 1.633483 | -0.87953 | 0.753957 | 3.77E-05 | 0.000723 |
| TXLNA | 1.121475 | -0.30486 | 0.816615 | 3.72E-05 | 0.000723 |
| MCF2L | 1.156151 | -0.20565 | 0.950502 | 3.88E-05 | 0.000741 |
| CACYBP | 1.514442 | -0.47318 | 1.041261 | 3.93E-05 | 0.000746 |
| GRAP2 | 1.49859 | -0.41466 | 1.083935 | 3.98E-05 | 0.000754 |
| CEP250 | 1.571708 | -0.62501 | 0.946695 | 4.04E-05 | 0.000759 |
| TDRD3 | 1.052819 | -0.18331 | 0.869507 | 4.10E-05 | 0.000767 |
| VAV3 | 1.625284 | -0.4668 | 1.158479 | 4.27E-05 | 0.000789 |
| WAS | 1.333557 | -0.26773 | 1.065825 | 4.27E-05 | 0.000789 |
| TBCC | 1.237824 | -0.42504 | 0.812787 | 4.39E-05 | 0.0008 |
| DAAM1 | 1.309303 | -0.35324 | 0.956065 | 4.45E-05 | 0.000803 |
| FGD3 | 1.239475 | -0.25358 | 0.985896 | 4.45E-05 | 0.000803 |
| NFX1 | 1.245681 | -0.60382 | 0.641858 | 4.51E-05 | 0.000804 |
| RAB27B | 1.358065 | -0.86656 | 0.491504 | 4.51E-05 | 0.000804 |
| STX8 | 1.007477 | -0.10885 | 0.898625 | 4.51E-05 | 0.000804 |
| PLCB2 | 1.732909 | -0.63745 | 1.095462 | 4.57E-05 | 0.000809 |
| CRADD | 1.156124 | -0.38147 | 0.77465 | 4.64E-05 | 0.000818 |
| CDC42BPB | 1.741518 | -0.60072 | 1.140801 | 4.70E-05 | 0.000819 |
| MINDY1 | 1.165432 | -0.29072 | 0.874711 | 4.67E-05 | 0.000819 |
| APPL2 | 1.60128 | -0.67542 | 0.925858 | 4.80E-05 | 0.00083 |
| CXCL5 | 1.145698 | 0.020432 | 1.16613 | 4.83E-05 | 0.000831 |
| GRIPAP1 | 1.297951 | -0.48077 | 0.817185 | 4.83E-05 | 0.000831 |
| CEP85 | 1.196988 | -0.41082 | 0.786169 | 5.18E-05 | 0.000868 |
| RAB11FIP3 | 1.498313 | -0.49523 | 1.003084 | 5.18E-05 | 0.000868 |
| GAK | 1.569166 | -1.55915 | 0.010016 | 5.25E-05 | 0.000874 |
| RFC4 | 1.023619 | -0.24107 | 0.782552 | 5.32E-05 | 0.000881 |
| ICA1 | 1.086554 | -0.17606 | 0.910497 | 5.39E-05 | 0.000885 |
| ATE1 | 1.313258 | -0.45312 | 0.860139 | 5.62E-05 | 0.0009 |

| Proteins | log2Fold Change | Log2 Average Control Group | Log2 Average Case Group | p.value | Adjusted_pval |
|---|---|---|---|---|---|
| SMOX | 1.023398 | -0.32613 | 0.697269 | 5.62E-05 | 0.0009 |
| STAT2 | 1.012572 | -0.27189 | 0.740685 | 5.62E-05 | 0.0009 |
| VPS54 | 1.278525 | -0.51155 | 0.766978 | 5.66E-05 | 0.000904 |
| PTPN6 | 1.182846 | -0.59522 | 0.587624 | 5.69E-05 | 0.000907 |
| UACA | 1.500015 | -0.45991 | 1.040101 | 5.85E-05 | 0.000927 |
| BECN1 | 1.007292 | -0.19002 | 0.817274 | 5.93E-05 | 0.000934 |
| DCTN1 | 1.040529 | -0.37607 | 0.664454 | 5.93E-05 | 0.000934 |
| CORO1A | 1.306183 | -0.21228 | 1.093901 | 6.01E-05 | 0.000936 |
| MIF | 1.043334 | -0.26213 | 0.781206 | 6.01E-05 | 0.000936 |
| USO1 | 1.125276 | -0.45083 | 0.674445 | 6.01E-05 | 0.000936 |
| CFAP410 | 1.131793 | -0.22297 | 0.908824 | 6.09E-05 | 0.000938 |
| HBS1L | 1.197034 | -0.30549 | 0.891541 | 6.09E-05 | 0.000938 |
| IMUP | 1.436864 | -0.37293 | 1.063938 | 6.09E-05 | 0.000938 |
| YARS1 | 2.02683 | -1.09881 | 0.928021 | 6.26E-05 | 0.00095 |
| BAG5 | 1.090134 | -0.2297 | 0.86043 | 6.35E-05 | 0.000955 |
| CMC1 | 1.269636 | -0.30262 | 0.967019 | 6.52E-05 | 0.000955 |
| EIF2S2 | 1.108317 | -0.4973 | 0.611016 | 6.43E-05 | 0.000955 |
| KIFC3 | 1.644073 | -0.71024 | 0.933834 | 6.35E-05 | 0.000955 |
| MAP3K5 | 1.589795 | -0.55061 | 1.039181 | 6.35E-05 | 0.000955 |
| PIKFYVE | 1.25085 | -0.42651 | 0.82434 | 6.52E-05 | 0.000955 |
| TARBP2 | 1.253248 | -0.2502 | 1.003048 | 6.61E-05 | 0.000965 |
| NEK7 | 1.124099 | -0.57783 | 0.546273 | 6.65E-05 | 0.000969 |
| MPP7 | 1.09827 | -0.30719 | 0.791076 | 6.79E-05 | 0.000981 |
| TOMM34 | 1.327083 | -0.70118 | 0.625898 | 6.88E-05 | 0.000992 |
| DLG4 | 1.231931 | -0.23099 | 1.000946 | 6.98E-05 | 0.000995 |
| FAF2 | 1.695916 | -0.22132 | 1.474599 | 6.98E-05 | 0.000995 |
| SMAD2 | 1.031623 | -0.15727 | 0.874354 | 6.98E-05 | 0.000995 |
| DOK1 | 1.651279 | -0.78628 | 0.865002 | 7.17E-05 | 0.001003 |
| EDF1 | 1.166118 | -0.19247 | 0.973647 | 7.17E-05 | 0.001003 |
| GCC1 | 1.57414 | -0.59671 | 0.97743 | 7.17E-05 | 0.001003 |
| SH3BP5 | 1.161896 | -0.31447 | 0.847422 | 7.26E-05 | 0.001007 |
| LIMD1 | 1.129346 | -0.22921 | 0.900141 | 7.46E-05 | 0.001026 |
| IST1 | 1.043623 | -0.38815 | 0.655471 | 7.56E-05 | 0.001027 |
| UMAD1 | 1.318588 | -0.30215 | 1.01644 | 7.56E-05 | 0.001027 |
| PTGES3L | 1.156135 | -0.24287 | 0.913265 | 7.61E-05 | 0.001031 |
| CA13 | 1.360273 | -0.36381 | 0.996461 | 7.66E-05 | 0.001033 |
| FCHO2 | 1.196928 | -0.25254 | 0.944391 | 7.77E-05 | 0.001039 |
| NFE2 | 1.323726 | -0.35496 | 0.96877 | 7.77E-05 | 0.001039 |
| HOMER2 | 1.270183 | -0.252 | 1.018185 | 8.08E-05 | 0.001063 |
| CLASP1 | 1.048825 | -0.14413 | 0.904698 | 8.19E-05 | 0.001067 |

| Proteins | log2Fold Change | Log2 Average Control Group | Log2 Average Case Group | p.value | Adjusted_pval |
|---|---|---|---|---|---|
| RIMOC1 | 1.156564 | -0.37456 | 0.782003 | 8.19E-05 | 0.001067 |
| TMED8 | 1.312653 | -0.41254 | 0.900108 | 8.19E-05 | 0.001067 |
| CDV3 | 1.040892 | -0.11376 | 0.927135 | 8.41E-05 | 0.001076 |
| SPAST | 1.446418 | -0.17788 | 1.268538 | 8.41E-05 | 0.001076 |
| TADA3 | 1.133733 | -0.25851 | 0.875219 | 8.41E-05 | 0.001076 |
| PYM1 | 1.601742 | -0.28231 | 1.319427 | 8.53E-05 | 0.001087 |
| CEP44 | 1.389109 | -0.52638 | 0.862728 | 8.76E-05 | 0.001096 |
| CRKL | 1.233495 | -0.41896 | 0.81453 | 8.76E-05 | 0.001096 |
| HPSE | 1.097204 | -0.24139 | 0.855811 | 8.76E-05 | 0.001096 |
| RASGRP2 | 1.594926 | -0.75069 | 0.844235 | 8.87E-05 | 0.001108 |
| SARG | 1.289173 | -0.2777 | 1.011475 | 8.99E-05 | 0.001118 |
| FHIT | 1.18953 | -0.37241 | 0.817116 | 9.11E-05 | 0.001128 |
| TBCB | 1.579847 | -0.7656 | 0.814249 | 9.11E-05 | 0.001128 |
| CEP170 | 1.154482 | -0.3213 | 0.833179 | 9.24E-05 | 0.001135 |
| VASP | 1.125417 | -0.22367 | 0.901746 | 9.24E-05 | 0.001135 |
| PPIB | 1.020634 | -0.14595 | 0.874688 | 9.48E-05 | 0.001152 |
| PPP1R2B | 1.221734 | -0.33723 | 0.884508 | 9.48E-05 | 0.001152 |
| SHROOM4 | 1.003355 | -0.54938 | 0.453977 | 9.48E-05 | 0.001152 |
| TPD52L2 | 1.195142 | -0.37078 | 0.82436 | 9.48E-05 | 0.001152 |
| ACOT13 | 1.165291 | -0.22094 | 0.944354 | 9.61E-05 | 0.00116 |
| COMT | 1.042965 | -0.26072 | 0.782243 | 9.61E-05 | 0.00116 |
| DIABLO | 1.294937 | -0.29759 | 0.997351 | 9.74E-05 | 0.001173 |
| ABRAXAS2 | 1.207431 | -0.58279 | 0.62464 | 1.00E-04 | 0.001194 |
| APLF | 1.270712 | -0.53475 | 0.735966 | 1.00E-04 | 0.001194 |
| GPX1 | 1.071353 | -0.20426 | 0.867092 | 1.00E-04 | 0.001194 |
| PNMA1 | 1.143102 | -0.45797 | 0.685132 | 0.000104 | 0.001226 |
| HARS1 | 1.005176 | -0.24599 | 0.759184 | 0.000107 | 0.001247 |
| PDLIM7 | 1.516809 | -0.33717 | 1.179641 | 0.000107 | 0.001247 |
| SRC | 1.322888 | -0.50482 | 0.818068 | 0.000107 | 0.001247 |
| PTPN11 | 1.212241 | -0.13778 | 1.074464 | 0.000108 | 0.001253 |
| RHOC | 1.025573 | -0.40243 | 0.623141 | 0.000108 | 0.001253 |
| SPART | 1.119963 | -0.24127 | 0.878688 | 0.000108 | 0.001253 |
| SESTD1 | 1.161573 | -0.66876 | 0.492814 | 0.00011 | 0.001267 |
| SNAP29 | 1.265563 | -0.40245 | 0.863109 | 0.000111 | 0.00127 |
| TWF2 | 1.200011 | -0.20249 | 0.997518 | 0.000111 | 0.00127 |
| SAMD14 | 1.622809 | -0.66691 | 0.955895 | 0.000114 | 0.001296 |
| CCDC43 | 1.157337 | -0.26938 | 0.887957 | 0.000116 | 0.001305 |
| FARSA | 1.480448 | -0.48647 | 0.993979 | 0.000116 | 0.001305 |
| CFAP36 | 1.013617 | -0.21953 | 0.79409 | 0.000119 | 0.00132 |
| NAA80 | 1.368516 | -0.30768 | 1.060838 | 0.000119 | 0.00132 |

| Proteins | log2Fold Change | Log2 Average Control Group | Log2 Average Case Group | p.value | Adjusted_pval |
|---|---|---|---|---|---|
| PRDX5 | 1.043742 | -0.33228 | 0.711461 | 0.000119 | 0.00132 |
| SAMD9L | 1.062163 | -0.26134 | 0.800818 | 0.000119 | 0.00132 |
| GIPC3 | 1.229032 | -0.61193 | 0.617106 | 0.00012 | 0.001329 |
| PDZD2 | 1.144348 | -0.32887 | 0.815475 | 0.00012 | 0.001329 |
| PDAP1 | 1.20789 | -0.24688 | 0.96101 | 0.000125 | 0.001377 |
| IRAG2 | 1.213666 | -0.26218 | 0.951486 | 0.00013 | 0.001414 |
| SUGP1 | 1.115014 | -0.54619 | 0.568824 | 0.00013 | 0.001414 |
| DMD | 1.010103 | -0.11879 | 0.891314 | 0.000133 | 0.001446 |
| MKNK1 | 1.118289 | -0.35163 | 0.766657 | 0.000137 | 0.001469 |
| NTPCR | 1.236894 | -0.32046 | 0.916435 | 0.000137 | 0.001469 |
| RGS10 | 1.182087 | -0.40036 | 0.781724 | 0.000137 | 0.001469 |
| DGKI | 1.169637 | -0.30253 | 0.867111 | 0.000139 | 0.00148 |
| IRF3 | 1.15368 | -0.32655 | 0.827135 | 0.00014 | 0.00149 |
| ITGB1BP2 | 1.361653 | -0.54772 | 0.813938 | 0.00014 | 0.00149 |
| RGS6 | 1.574515 | -0.61446 | 0.960053 | 0.000144 | 0.00152 |
| DOK2 | 1.490416 | -0.39647 | 1.093942 | 0.000146 | 0.001528 |
| ASRGL1 | 1.016962 | -0.15385 | 0.863113 | 0.000148 | 0.001542 |
| VPS4B | 1.279297 | -0.15661 | 1.122686 | 0.000148 | 0.001542 |
| NHERF1 | 1.045588 | -0.14549 | 0.900095 | 0.00015 | 0.001559 |
| CEP135 | 1.273536 | -0.50393 | 0.769606 | 0.000151 | 0.001566 |
| SKAP2 | 1.434847 | -0.65161 | 0.78324 | 0.000158 | 0.001608 |
| UFD1 | 1.501222 | -0.28124 | 1.219984 | 0.000158 | 0.001608 |
| KIAA0513 | 1.533446 | -0.58655 | 0.946894 | 0.000162 | 0.001631 |
| TCF25 | 1.233839 | -0.16944 | 1.064402 | 0.000162 | 0.001631 |
| CNP | 1.026909 | -0.28801 | 0.738904 | 0.000168 | 0.001676 |
| RENBP | 1.323389 | -0.47525 | 0.848138 | 0.000173 | 0.00171 |
| EIF4E | 1.916788 | -0.85127 | 1.065518 | 0.000175 | 0.001726 |
| PELO | 1.282977 | -0.45309 | 0.829886 | 0.000177 | 0.001742 |
| TRAF3IP1 | 1.185581 | -0.9552 | 0.230381 | 0.000182 | 0.001781 |
| HSPB1 | 1.269246 | -0.49255 | 0.776697 | 0.000184 | 0.0018 |
| GGA3 | 1.324189 | -0.39718 | 0.927011 | 0.000186 | 0.00182 |
| SIRT2 | 1.087534 | -0.28314 | 0.804391 | 0.000198 | 0.001915 |
| SNX29 | 1.528797 | -0.66436 | 0.864434 | 0.000198 | 0.001915 |
| MAGEB2 | 1.04782 | -0.16437 | 0.883446 | 0.000201 | 0.001929 |
| ZFYVE19 | 1.070896 | -0.25573 | 0.815161 | 0.000204 | 0.001947 |
| C2orf88 | 1.253361 | -0.3132 | 0.940164 | 0.000205 | 0.001956 |
| PDLIM5 | 1.106053 | -0.38698 | 0.719074 | 0.000206 | 0.001958 |
| BCL2L1 | 1.524752 | -0.50777 | 1.016984 | 0.000209 | 0.001976 |
| PFKFB2 | 1.00266 | -0.26424 | 0.738424 | 0.000211 | 0.001987 |
| TAB2 | 1.328065 | -0.51267 | 0.815392 | 0.000213 | 0.001996 |

| Proteins | log2Fold Change | Log2 Average Control Group | Log2 Average Case Group | p.value | Adjusted_pval |
|---|---|---|---|---|---|
| TMF1 | 1.21868 | -0.27924 | 0.939445 | 0.000217 | 0.002021 |
| NDUFB7 | 1.002808 | -0.10463 | 0.89818 | 0.00022 | 0.002036 |
| EHD3 | 1.331588 | -0.57824 | 0.753345 | 0.000225 | 0.00207 |
| PRXL2B | 1.298598 | -0.3651 | 0.933494 | 0.000225 | 0.00207 |
| PRKG1 | 1.558113 | -0.60342 | 0.954688 | 0.000228 | 0.002086 |
| SNAP23 | 1.219172 | -0.47827 | 0.740903 | 0.000228 | 0.002086 |
| SCAMP3 | 1.036691 | -0.10242 | 0.934274 | 0.000231 | 0.002098 |
| DTD1 | 1.421308 | -0.52258 | 0.898731 | 0.000234 | 0.002114 |
| CACNB3 | 1.127611 | -0.37968 | 0.747931 | 0.000237 | 0.002123 |
| OPLAH | 1.090651 | -0.41097 | 0.679683 | 0.000237 | 0.002123 |
| RILPL2 | 1.271225 | -0.54839 | 0.722833 | 0.00024 | 0.002136 |
| CDC37 | 1.318617 | -0.23066 | 1.087957 | 0.000243 | 0.002155 |
| MGMT | 1.213929 | -0.37833 | 0.835597 | 0.000246 | 0.002179 |
| ABLIM3 | 1.127397 | -0.2693 | 0.858092 | 0.000252 | 0.00222 |
| FYB1 | 1.270117 | -0.46467 | 0.805443 | 0.000252 | 0.00222 |
| OTUD6B | 1.0162 | -0.3192 | 0.697001 | 0.000259 | 0.002265 |
| EIF4G1 | 1.282087 | -0.56747 | 0.714616 | 0.000265 | 0.002293 |
| PPP1R9B | 1.145528 | -0.41024 | 0.735289 | 0.000265 | 0.002293 |
| ASAP2 | 1.227251 | -0.55392 | 0.673336 | 0.000268 | 0.002296 |
| CDKN1A | 1.204732 | -0.4278 | 0.776929 | 0.000272 | 0.002313 |
| STK4 | 1.080741 | -0.41172 | 0.669025 | 0.000272 | 0.002313 |
| CAMLG | 1.13686 | -0.29983 | 0.837026 | 0.000279 | 0.002353 |
| CC2D1A | 1.034342 | -0.14233 | 0.892007 | 0.000282 | 0.002364 |
| REPS2 | 1.034629 | -0.39492 | 0.639712 | 0.000286 | 0.002383 |
| TBC1D23 | 1.102017 | -0.21829 | 0.883725 | 0.000287 | 0.002394 |
| FMNL3 | 1.163534 | -0.47764 | 0.685893 | 0.000296 | 0.002443 |
| HDAC6 | 1.032828 | -0.70003 | 0.332795 | 0.000311 | 0.002555 |
| CSDE1 | 1.08691 | -0.16362 | 0.923287 | 0.000327 | 0.00266 |
| ELAC1 | 1.059931 | -0.50542 | 0.554507 | 0.000331 | 0.002681 |
| TACC3 | 1.316461 | -0.47688 | 0.839581 | 0.000335 | 0.002706 |
| NT5C3A | 1.329958 | -0.53487 | 0.795088 | 0.000339 | 0.002727 |
| LDLRAP1 | 1.304548 | -0.68073 | 0.623823 | 0.000344 | 0.002732 |
| IQCE | 1.222686 | -0.47375 | 0.748933 | 0.000361 | 0.002857 |
| DTYMK | 1.10836 | -0.41268 | 0.695679 | 0.00037 | 0.002915 |
| UBXN6 | 1.049547 | -0.41815 | 0.631398 | 0.00037 | 0.002915 |
| DENND2C | 1.002777 | -0.22485 | 0.777929 | 0.000388 | 0.003004 |
| ERBIN | 1.111926 | -0.10654 | 1.005386 | 0.000388 | 0.003004 |
| PIBF1 | 1.001877 | -0.2156 | 0.786277 | 0.000388 | 0.003004 |
| SMTN | 1.188769 | -0.295 | 0.893766 | 0.000388 | 0.003004 |
| PPP1R12A | 1.185323 | -0.1258 | 1.05952 | 0.000391 | 0.003018 |

| Proteins | log2Fold Change | Log2 Average Control Group | Log2 Average Case Group | p.value | Adjusted_pval |
|---|---|---|---|---|---|
| NFU1 | 1.015549 | -0.11384 | 0.901707 | 0.000398 | 0.00306 |
| LCP2 | 1.339155 | -0.45302 | 0.886136 | 0.000403 | 0.003084 |
| PMVK | 1.384743 | -0.81829 | 0.566457 | 0.000403 | 0.003084 |
| TRIP11 | 1.549385 | -0.62523 | 0.924152 | 0.000407 | 0.003104 |
| CASP7 | 1.096223 | -0.22595 | 0.870274 | 0.000417 | 0.003163 |
| BICD2 | 1.021877 | -0.23 | 0.791882 | 0.000428 | 0.003222 |
| RAPGEF2 | 1.111408 | -0.33406 | 0.777346 | 0.000428 | 0.003222 |
| VTA1 | 1.008648 | -0.34212 | 0.666524 | 0.000443 | 0.003313 |
| MYDGF | 1.034915 | -0.01765 | 1.017261 | 0.000465 | 0.003448 |
| ERP29 | 1.040492 | -0.08905 | 0.951444 | 0.000471 | 0.00348 |
| HNRNPK | 1.153333 | -0.2706 | 0.882732 | 0.000471 | 0.00348 |
| EBAG9 | 1.058765 | -0.13762 | 0.921148 | 0.000482 | 0.00355 |
| TOM1L2 | 1.189334 | -0.8412 | 0.348131 | 0.000482 | 0.00355 |
| PPFIBP1 | 1.073758 | -0.25225 | 0.821504 | 0.000488 | 0.003579 |
| SUGT1 | 1.034761 | -0.20798 | 0.826782 | 0.000494 | 0.003612 |
| FLI1 | 1.37396 | -0.43593 | 0.938035 | 0.000553 | 0.003949 |
| SV2A | 1.043555 | -0.07923 | 0.964324 | 0.000563 | 0.003999 |
| FAM221A | 1.067129 | -0.30431 | 0.762823 | 0.000573 | 0.00406 |
| CLIP2 | 1.213663 | -0.53794 | 0.675722 | 0.000584 | 0.004112 |
| MESD | 1.328162 | -0.34681 | 0.981347 | 0.000638 | 0.004406 |
| TBCA | 1.274013 | -0.23012 | 1.043893 | 0.000641 | 0.004415 |
| MIF4GD | 1.02561 | -0.32317 | 0.702443 | 0.000649 | 0.004445 |
| PHACTR2 | 1.245193 | -0.31585 | 0.92934 | 0.000649 | 0.004445 |
| EIF1AX | 1.147399 | -0.36725 | 0.780152 | 0.000657 | 0.00448 |
| GMPR2 | 1.066742 | -0.22873 | 0.83801 | 0.000657 | 0.00448 |
| IQUB | 1.329137 | -1.30178 | 0.027361 | 0.000713 | 0.004773 |
| STK24 | 1.049957 | -0.10171 | 0.948244 | 0.000721 | 0.004817 |
| CIRBP | 1.244165 | -0.51069 | 0.733472 | 0.000755 | 0.004985 |
| DDX60 | 1.069572 | -0.38026 | 0.689309 | 0.000751 | 0.004985 |
| RBPMS2 | 1.147493 | -0.39696 | 0.750533 | 0.000764 | 0.005007 |
| GOLGA1 | 1.445032 | -0.4812 | 0.963836 | 0.000778 | 0.005064 |
| ARHGEF1 | 1.01611 | -0.27535 | 0.740759 | 0.000791 | 0.005134 |
| DNM1 | 1.192114 | -0.44953 | 0.742586 | 0.00081 | 0.00518 |
| RPAP3 | 1.036565 | -0.28789 | 0.748677 | 0.00081 | 0.00518 |
| GRK5 | 1.012712 | -0.29289 | 0.719822 | 0.000833 | 0.005319 |
| ESYT2 | 1.179241 | -0.42678 | 0.752457 | 0.000888 | 0.005634 |
| RRAS | 1.325219 | -0.16687 | 1.158345 | 0.000919 | 0.005797 |
| DNAJB1 | 1.029184 | -0.31966 | 0.709522 | 0.000951 | 0.005945 |
| PDLIM1 | 1.160866 | -0.43111 | 0.729756 | 0.00114 | 0.006814 |
| STX16 | 1.022245 | -0.36918 | 0.653065 | 0.001206 | 0.007145 |

| Proteins | log2Fold Change | Log2 Average Control Group | Log2 Average Case Group | p.value | Adjusted_pval |
|---|---|---|---|---|---|
| SYK | 1.097773 | -0.49174 | 0.60603 | 0.00122 | 0.007218 |
| CDR2L | 1.105064 | -0.3393 | 0.765768 | 0.00129 | 0.007559 |
| CEP131 | 1.280123 | -0.35157 | 0.928555 | 0.001364 | 0.007907 |
| PPP4R4 | 1.081123 | -0.50795 | 0.57317 | 0.001364 | 0.007907 |
| AP1G2 | 1.152572 | -0.54046 | 0.612111 | 0.001442 | 0.008287 |
| DAB2 | 1.13309 | -0.31493 | 0.818163 | 0.001507 | 0.00858 |
| CCDC14 | 1.103792 | -0.59312 | 0.510673 | 0.001558 | 0.008804 |
| SERPINH1 | 1.001675 | -0.26106 | 0.740615 | 0.001645 | 0.00912 |
| MINK1 | 1.226591 | -0.70165 | 0.524942 | 0.001937 | 0.010481 |
| SMAD1 | 1.060591 | -0.32788 | 0.732713 | 0.001937 | 0.010481 |
| NSFL1C | 1.022292 | -0.25798 | 0.764307 | 0.002134 | 0.011411 |
| SLA2 | 1.035371 | -0.14878 | 0.886595 | 0.002204 | 0.011726 |
| RGS22 | 1.182625 | -1.36404 | -0.18141 | 0.002276 | 0.012025 |
| FRMD4B | 1.217243 | -0.52237 | 0.694873 | 0.002585 | 0.013346 |
| FKBP1B | 1.339256 | -0.39707 | 0.942182 | 0.002696 | 0.013816 |
| PNP | 1.325501 | -0.38186 | 0.943643 | 0.003056 | 0.015288 |
| HDAC5 | 1.011247 | -0.10882 | 0.902425 | 0.00368 | 0.017832 |
| DNAJB13 | 1.133394 | -1.02371 | 0.109683 | 0.004598 | 0.021532 |
| SNCA | 1.267568 | -0.43031 | 0.837262 | 0.004931 | 0.022933 |
| GOLGA8CP | 1.059708 | -1.43759 | -0.37788 | 0.005608 | 0.0253 |
| TECPR1 | 1.176118 | -1.56077 | -0.38465 | 0.005663 | 0.025465 |

**Supplementary Table 2. Canonical Pathways**

This table provides canonical pathways in which significantly dysregulated proteins were enriched. Pathways with -log(p-value) greater than 1.3 and |z-score| greater than or equal to 2 were listed here. Z-score was calculated based on the direction of expression changes of proteins in our dataset relative to the curated literature-defined direction of effects in that pathway, in accordance with the Ingenuity Pathway Analysis (IPA) algorithm. Z-scores greater than 2 indicate predicted activation, whereas those less than -2 indicate predicted inhibition.

| Ingenuity Canonical Pathways | -log (p-value) | Ratio | z-score | Molecules |
|---|---|---|---|---|
| RHO GTPase cycle | 8.22 | 0.0622 | 5.292 | ARHGAP10, ARHGAP35, ARHGAP45, ARHGEF1, ARHGEF12, BCR, CDC37, CDC42BPB, DAAM1, ERBIN, ESYT1, FAF2, FGD3, FMNL3, GIT1, GOLGA3, GOPC, MCF2L, MPP7, NSFL1C, OPHN1, RHOC, SNAP23, SRC, UACA, VAV3, WAS, WASF1 |
| GPVI-mediated activation cascade | 5.72 | 0.2 | 2.646 | CLEC1B, LCP2, MPIG6B, PTPN11, PTPN6, SYK, VAV3 |
| Signaling by ERBB2 | 5.68 | 0.16 | 2.828 | AKT2, CDC37, EGF, ERBIN, RNF41, SRC, USP8, YES1 |
| TNFR1 Signaling | 5.68 | 0.16 | 2.646 | CASP2, CASP3, CASP7, CHUK, CRADD, FADD, IKBKG, TRAF2 |
| Role of PKR in Interferon Induction and Antiviral Response | 5.49 | 0.0909 | 2.714 | BAX, CASP3, CHUK, EIF2AK2, FADD, IKBKG, IRF3, MAP2K6, MAVS, STAT2, TAB2, TARBP2 |
| FLT3 Signaling | 5.46 | 0.184 | 2.646 | AKT2, FOXO3, GRAP2, PTPN11, SLA2, STAT5B, SYK |
| PKR-mediated signaling | 5.37 | 0.109 | 3.162 | CHUK, EIF2AK2, EIF2B4, EIF2S2, IKBKG, MAP2K6, MAVS, SNCA, TARBP2, TRIM25 |
| NLR signaling pathways | 5.3 | 0.143 | 2.828 | BCL2L1, CASP2, CHUK, IKBKG, IRAK1, MAP2K6, SUGT1, TAB2 |
| Signal regulatory protein family interactions | 5.25 | 0.312 | 2.236 | FYB1, PTPN11, PTPN6, SKAP2, SRC |
| Chronic Myeloid Leukemia Signaling | 5.24 | 0.0625 | 2.5 | AKT2, AXIN1, BCL2L1, CDKN1A, CHUK, CRKL, FOXO3, HDAC5, HDAC6, IKBKG, NFATC1, PCBP2, PLCB2, PTPN11, PTPN6, RRAS, STAT5B |
| Role of RIG1-like Receptors in Antiviral Innate Immunity | 5.02 | 0.159 | 2.646 | CHUK, FADD, IKBKG, IRF3, MAVS, TRAF2, TRIM25 |

| Ingenuity Canonical Pathways | -log (p-value) | Ratio | z-score | Molecules |
|---|---|---|---|---|
| DDX58/IFIH1-mediated induction of interferon-alpha/beta | 4.97 | 0.111 | 3 | CHUK, FADD, IKBKG, IRF3, MAVS, PCBP2, TAX1BP1, TRAF2, TRIM25 |
| Cilium Assembly | 4.87 | 0.0686 | 3.742 | CEP131, CEP135, CEP250, CETN2, CKAP5, CLASP1, DCTN1, HDAC6, NEDD1, RAB11FIP3, RAB3IP, SDCCAG8, TRAF3IP1, TRIP11 |
| Role of Osteoclasts in Rheumatoid Arthritis Signaling Pathway | 4.78 | 0.0576 | 2.5 | AKT2, CHUK, FOXO1, FOXO3, IKBKG, LCP2, MAP2K6, MAP3K5, NFATC1, PTPN1, RHOC, RRAS, SRC, SYK, TAB2, TRAF2, VAV3 |
| Cachexia Signaling Pathway | 4.74 | 0.0528 | 2.524 | AKT2, BECN1, CASP2, CASP3, CASP7, CD40LG, CHUK, EIF2AK2, EIF2AK3, EIF2B4, EIF2S2, FOXO1, FOXO3, IKBKG, INSL3, PRKAR1A, SMAD2, STAT2, STAT5B |
| Endoplasmic Reticulum Stress Pathway | 4.62 | 0.238 | 2.236 | CASP3, CASP7, EIF2AK3, MAP3K5, TRAF2 |
| RANK Signaling in Osteoclasts | 4.59 | 0.1 | 3 | AKT2, CHUK, IKBKG, MAP2K6, MAP3K5, NFATC1, SRC, TAB2, TRAF2 |
| Transcriptional regulation by RUNX2 | 4.59 | 0.137 | 2.646 | AKT2, BAX, CDKN1A, HDAC6, SMAD1, SRC, YES1 |
| Neuregulin Signaling | 4.51 | 0.087 | 2.121 | AKT2, CRKL, DLG4, EGF, ERBIN, PTPN11, RNF41, RRAS, SRC, STAT5B |
| Fcγ Receptor-mediated Phagocytosis in Macrophages and Monocytes | 4.48 | 0.0968 | 3 | AKT2, FYB1, LCP2, SRC, SYK, VASP, VAV3, WAS, YES1 |
| Lymphotoxin β Receptor Signaling | 4.42 | 0.13 | 2.449 | AKT2, BCL2L1, CASP3, CHUK, DIABLO, IKBKG, TRAF2 |
| Fc Epsilon RI Signaling | 4.41 | 0.0847 | 2.333 | AKT2, GRAP2, INPPL1, LCP2, MAP2K6, PLA2G4A, PTPN11, RRAS, SYK, VAV3 |
| Intrinsic Pathway for Apoptosis | 4.37 | 0.127 | 2.646 | AKT2, BAX, BCL2L1, CASP3, CASP7, DIABLO, UACA |
| Interleukin-3, Interleukin-5 and GM-CSF signaling | 4.37 | 0.127 | 2.646 | CRKL, INPPL1, PTPN11, PTPN6, STAT5B, SYK, YES1 |

| Ingenuity Canonical Pathways | -log (p-value) | Ratio | z-score | Molecules |
|---|---|---|---|---|
| MyD88-independent TLR4 cascade | 4.36 | 0.158 | 2.449 | CHUK, FADD, IKBKG, IRF3, PTPN11, TAB2 |
| Myelination Signaling Pathway | 4.32 | 0.053 | 2.183 | AKT2, ARHGAP35, AXIN1, CNP, EIF4E, HDAC5, HDAC6, NFATC1, PRKAR1A, PTPN11, RAPGEF2, RRAS, SMAD1, SMAD2, SRC, TSC1, YES1 |
| p38 MAPK Signaling | 4.32 | 0.0826 | 3 | FADD, HSPB1, IRAK1, IRAK4, MAP2K6, MAP3K5, MKNK1, PLA2G4A, TAB2, TRAF2 |
| Role of Tissue Factor in Cancer | 4.28 | 0.0644 | 2.496 | AKT2, BCL2L1, CASP3, CHUK, EGF, EIF4E, IKBKG, MAP2K6, PTPN11, RRAS, SRC, STAT5B, YES1 |
| MyD88 cascade initiated on plasma membrane | 4.23 | 0.2 | 2.236 | CHUK, IKBKG, IRAK1, IRAK4, TAB2 |
| Necroptosis Signaling Pathway | 4.16 | 0.0724 | 2.714 | CHUK, EIF2AK2, FADD, IKBKG, IRF3, PLA2G4A, STAT2, TAB2, TNIP1, TOMM34, TRAF2 |
| p75 NTR receptor-mediated signalling | 4.06 | 0.0705 | 3.317 | ARHGEF1, ARHGEF12, CASP2, CASP3, FGD3, IRAK1, MCF2L, STK24, STK39, STK4, VAV3 |
| Signaling by SCF-KIT | 4.04 | 0.14 | 2.449 | GRAP2, PTPN11, PTPN6, SRC, STAT5B, YES1 |
| Activation of IRF by Cytosolic Pattern Recognition Receptors | 3.98 | 0.111 | 2.646 | CHUK, FADD, IKBKG, IRF3, MAVS, PPIB, STAT2 |
| NAFLD Signaling Pathway | 3.98 | 0.0602 | 2.496 | AKT2, BAX, CASP3, CASP7, CD40LG, CHUK, EIF2AK3, FOXO1, FOXO3, GYS1, IKBKG, MAP3K5, TRAF2 |
| Systemic Lupus Erythematosus in B Cell Signaling Pathway | 3.86 | 0.0441 | 2.236 | AKT2, BCL2L1, CD40LG, FOXO1, FOXO3, INPPL1, IRAK1, IRAK4, IRF3, MAVS, NFATC1, PTPN11, PTPN6, RASGRP2, RRAS, SRC, STAT2, SYK, TRAF2, YES1 |
| Signaling by NTRK3 (TRKC) | 3.83 | 0.167 | 2.236 | BAX, CASP2, CASP3, CASP7, SRC |
| FOXO-mediated transcription of cell cycle genes | 3.76 | 0.235 | 2 | CDKN1A, FOXO1, FOXO3, SMAD2 |
| Signaling by CSF1 (M-CSF) in myeloid cells | 3.76 | 0.161 | 2.236 | GRAP2, INPPL1, PTPN11, SRC, YES1 |

| Ingenuity Canonical Pathways | -log (p-value) | Ratio | z-score | Molecules |
|---|---|---|---|---|
| MyD88 dependent cascade initiated on endosome | 3.76 | 0.161 | 2.236 | CHUK, IKBKG, IRAK1, IRAK4, TAB2 |
| Intra-Golgi and retrograde Golgi-to-ER traffic | 3.63 | 0.0588 | 3.464 | BICD2, DCTN1, DCTN6, GCC1, GOLGA1, KIF22, PLA2G4A, SNAP29, STX16, TMF1, TRIP11, VPS54 |
| Toll Like Receptor 3 (TLR3) Cascade | 3.62 | 0.152 | 2.236 | CHUK, FADD, IKBKG, IRF3, TAB2 |
| PIP3 activates AKT signaling | 3.61 | 0.068 | 3.162 | AKT2, CDKN1A, CHUK, EGF, FOXO1, FOXO3, IRAK1, IRAK4, PTPN11, SRC |
| Mitotic Prometaphase | 3.61 | 0.0585 | 3.464 | CEP131, CEP135, CEP250, CETN2, CKAP5, CLASP1, DCTN1, EML4, MZT2B, NEDD1, NEK7, SDCCAG8 |
| Small Cell Lung Cancer Signaling | 3.59 | 0.0833 | 2.236 | AKT2, BCL2L1, CHUK, FHIT, HDAC5, HDAC6, IKBKG, TRAF2 |
| Mitotic G2-G2/M phases | 3.57 | 0.058 | 3.464 | CDKN1A, CEP131, CEP135, CEP250, CETN2, CKAP5, CLASP1, DCTN1, MZT2B, NEDD1, PPP1R12A, SDCCAG8 |
| IL-8 Signaling | 3.57 | 0.058 | 2.714 | AKT2, BAX, BCL2L1, CHUK, IKBKG, IRAK1, IRAK4, PLCB2, RHOC, RRAS, SRC, VASP |
| RHO GTPases activate CIT | 3.56 | 0.211 | 2 | DLG4, MYH9, PPP1R12A, RHOC |
| Modulation of host responses by IFN-stimulated genes | 3.56 | 0.211 | 2 | CHUK, EIF2AK3, IKBKG, TRIM25 |
| ISG15 antiviral mechanism | 3.54 | 0.0946 | 2.646 | BECN1, EIF2AK2, EIF4E, EIF4G1, EIF4G3, IRF3, TRIM25 |
| NOD1/2 Signaling Pathway | 3.53 | 0.0615 | 2.53 | ATG16L1, CD40LG, CHUK, ERBIN, IKBKG, IRF3, MAP2K6, MAVS, SUGT1, TAB2, TRAF2 |
| Protein Sorting Signaling Pathway | 3.53 | 0.0615 | 3.317 | AP1G2, GCC1, GGA3, GOLGA1, PRKAR1A, SERPINH1, SNX29, SRC, TBC1D23, TMED8, YES1 |
| Actin Cytoskeleton Signaling | 3.5 | 0.0537 | 2.309 | ARHGAP35, ARHGEF1, ARHGEF12, CRKL, EGF, FGD3, GIT1, MYH9, PPP1R12A, RRAS, VAV3, WAS, WASF1 |

| Ingenuity Canonical Pathways | -log (p-value) | Ratio | z-score | Molecules |
|---|---|---|---|---|
| Insulin Secretion Signaling Pathway | 3.44 | 0.0504 | 3.051 | CACNB3, EIF2AK3, EIF2B4, EIF2S2, EIF4E, EIF4G1, EIF4G3, PLCB2, PRKAR1A, SRC, STAT2, STAT5B, STX16, YES1 |
| Toll-like Receptor Signaling | 3.43 | 0.0909 | 2.449 | CHUK, EIF2AK2, IKBKG, IRAK1, IRAK4, MAP2K6, TAB2 |
| Co-inhibition by CTLA4 | 3.38 | 0.19 | 2 | AKT2, PTPN11, SRC, YES1 |
| TNF signaling | 3.36 | 0.105 | 2.449 | CHUK, FADD, IKBKG, TAB2, TAX1BP1, TRAF2 |
| Deubiquitination | 3.3 | 0.0488 | 3.742 | ABRAXAS2, AXIN1, BECN1, IKBKG, MAVS, SMAD1, SMAD2, TADA3, TNIP1, TRAF2, TRIM25, UFD1, USP25, USP8 |
| MAP kinase activation | 3.28 | 0.128 | 2.236 | CHUK, IKBKG, IRAK1, MAP2K6, TAB2 |
| Immunogenic Cell Death Signaling Pathway | 3.2 | 0.0833 | 2.646 | ATG16L1, BAX, BECN1, CASP3, EIF2AK3, FADD, IRF3 |
| Natural Killer Cell Signaling | 3.2 | 0.0561 | 2.111 | AKT2, IRAK4, LCP2, MAP3K5, NFATC1, PTPN11, PTPN6, RRAS, SYK, VAV3, WAS |
| RET signaling | 3.17 | 0.122 | 2.236 | DOK1, DOK2, PDLIM7, PTPN11, SRC |
| Tumoricidal Function of Hepatic Natural Killer Cells | 3.15 | 0.167 | 2 | BAX, CASP3, CASP7, FADD |
| Reelin Signaling in Neurons | 3.15 | 0.0647 | 3 | AKT2, ARHGEF1, ARHGEF12, CRKL, MAP2K6, MAPK8IP3, SRC, WASF1, YES1 |
| MyD88:MAL(TIRAP) cascade initiated on plasma membrane | 3.13 | 0.119 | 2.236 | CHUK, IKBKG, IRAK1, IRAK4, TAB2 |
| Myocardin Signaling Pathway | 3.04 | 0.069 | 2.121 | AKT2, CDKN1A, EGF, FOXO3, HDAC5, MRTFA, SMAD1, SMAD2 |
| TAK1-dependent IKK and NF-kappa-B activation | 3.03 | 0.114 | 2.236 | CHUK, IKBKG, IRAK1, TAB2, TRAF2 |
| Integrin signaling | 2.95 | 0.148 | 2 | PTPN1, RASGRP2, SRC, SYK |
| MTOR signalling | 2.89 | 0.143 | 2 | AKT2, EIF4E, EIF4G1, TSC1 |

| Ingenuity Canonical Pathways | -log (p-value) | Ratio | z-score | Molecules |
|---|---|---|---|---|
| MicroRNA Biogenesis Signaling Pathway | 2.87 | 0.0546 | 2.333 | AKT2, CSDE1, DDX19B, DNAJB1, EGF, RRAS, SMAD1, SMAD2, TARBP2, ZC3H12A |
| Acute Phase Response Signaling | 2.85 | 0.0543 | 2.53 | AKT2, CHUK, HNRNPK, IKBKG, IRAK1, MAP2K6, MAP3K5, PTPN11, RRAS, TRAF2 |
| Role of Macrophages, Fibroblasts and Endothelial Cells in Rheumatoid Arthritis | 2.84 | 0.0437 | 3.051 | AKT2, AXIN1, CHUK, DAAM1, IKBKG, IRAK1, IRAK4, MAP2K6, MIF, NFATC1, PLCB2, RRAS, SRC, TRAF2 |
| IL-1 Signaling | 2.83 | 0.0722 | 2.646 | CHUK, IKBKG, IRAK1, IRAK4, MAP2K6, PRKAR1A, TAB2 |
| Interconversion of nucleotide di- and triphosphates | 2.83 | 0.138 | 2 | AK2, DCTD, DTYMK, RRM2B |
| TCR signaling | 2.78 | 0.063 | 2.828 | CHUK, FYB1, GRAP2, IKBKG, LCP2, TAB2, VASP, WAS |
| Glycation Signaling Pathway | 2.75 | 0.0493 | 2.714 | AKT2, CASP3, CD40LG, CHUK, EIF2AK3, IKBKG, IRAK1, IRAK4, RRAS, SNCA, SRC |
| IL-6 Signaling | 2.74 | 0.062 | 2.828 | AKT2, CHUK, HSPB1, IKBKG, MAP2K6, PTPN11, RRAS, TRAF2 |
| Senescence Pathway | 2.72 | 0.0442 | 2.887 | AKT2, CACNB3, CDKN1A, CHUK, EIF4E, FOXO3, IKBKG, IRF3, MAP2K6, NFATC1, RRAS, SMAD1, SMAD2 |
| Interleukin-1 family signaling | 2.72 | 0.0615 | 2.828 | CHUK, IKBKG, IRAK1, IRAK4, MAP2K6, PTPN11, PTPN6, TAB2 |
| Role of MAPK Signaling in Inhibiting the Pathogenesis of Influenza | 2.7 | 0.0789 | 2.449 | BAX, CASP3, EIF2AK2, MAP2K6, MAP3K5, PLA2G4A |
| NF-κB Signaling | 2.69 | 0.0407 | 3.357 | AKT2, AZI2, CD40LG, CHUK, EGF, EIF2AK2, FADD, IKBKG, IRAK1, IRAK4, MAP2K6, RRAS, TAB2, TNIP1, TRAF2 |
| Regulation of the Epithelial Mesenchymal Transition by Growth Factors Pathway | 2.68 | 0.0515 | 3.162 | AKT2, CD40LG, CHUK, EGF, FOXO1, IKBKG, MAP2K6, PTPN11, RRAS, SMAD2 |

| Ingenuity Canonical Pathways | -log (p-value) | Ratio | z-score | Molecules |
|---|---|---|---|---|
| Interferon alpha/beta signaling | 2.67 | 0.0779 | 2.449 | EIF2AK2, IRF3, PTPN1, PTPN11, PTPN6, STAT2 |
| CGAS-STING Signaling Pathway | 2.66 | 0.0602 | 2.828 | ATG16L1, BAX, CD40LG, CDKN1A, CHUK, EIF2AK3, IKBKG, IRF3 |
| Gα12/13 Signaling | 2.66 | 0.0602 | 2.828 | AKT2, ARHGEF1, CHUK, IKBKG, MAP3K5, RRAS, SRC, VAV3 |
| NF-κB Activation by Viruses | 2.64 | 0.0769 | 2.449 | AKT2, CHUK, EIF2AK2, IKBKG, RRAS, TRAF2 |
| Signaling by MET | 2.61 | 0.0759 | 2.449 | CRKL, GGA3, PTPN1, PTPN11, SRC, USP8 |
| Orexin Signaling Pathway | 2.58 | 0.047 | 2.111 | AKT2, CACNB3, CASP3, FOXO1, PIKFYVE, PLA2G4A, PLCB2, PRKAR1A, PTPN11, SMAD1, SRC |
| G alpha (12/13) signalling events | 2.58 | 0.075 | 2.449 | ARHGEF1, ARHGEF12, FGD3, MCF2L, RHOC, VAV3 |
| 4-1BB Signaling in T Lymphocytes | 2.57 | 0.118 | 2 | CHUK, IKBKG, MAP3K5, TRAF2 |
| FLT3 Signaling in Hematopoietic Progenitor Cells | 2.56 | 0.0741 | 2.449 | AKT2, EIF4E, PTPN11, RRAS, STAT2, STAT5B |
| Activin Inhibin Signaling Pathway | 2.54 | 0.0493 | 3.162 | AKT2, CDKN1A, CHUK, FOXO3, IKBKG, MAP2K6, NFATC1, SMAD1, SMAD2, TRAF2 |
| Role of MAPK Signaling in Promoting the Pathogenesis of Influenza | 2.52 | 0.0636 | 2.646 | BAX, BECN1, CASP3, MAP2K6, MAP3K5, PLA2G4A, RRAS |
| Synaptogenesis Signaling Pathway | 2.47 | 0.0414 | 3.464 | AKT2, CACNB3, CRKL, DLG4, PRKAR1A, RASGRP2, RRAS, SNCA, SRC, STX16, WAS, WASF1, YES1 |
| Integrin to Cytoskeleton Signaling Pathway | 2.47 | 0.0481 | 2.121 | AKT2, CRKL, GIT1, PIKFYVE, PPP1R12A, RHOC, RRAS, SRC, VASP, WAS |
| Glutaminergic Receptor Signaling Pathway (Enhanced) | 2.42 | 0.0408 | 3.606 | AKT2, CACNB3, CHUK, DGKA, DGKI, EIF4E, IKBKG, MKNK1, PLA2G4A, PLCB2, PRKAR1A, SRC, STX16 |
| tRNA Charging | 2.39 | 0.105 | 2 | FARSA, HARS1, MARS1, YARS1 |

| Ingenuity Canonical Pathways | -log (p-value) | Ratio | z-score | Molecules |
|---|---|---|---|---|
| Neutrophil degranulation | 2.38 | 0.0356 | 4.123 | ARHGAP45, CNN2, CPPED1, CRACR2A, DBNL, FAF2, GMFG, HPSE, IRAG2, IST1, MIF, PDAP1, PNP, PTPN6, SERPINB1, SNAP23, SNAP29 |
| IL-2 Signaling | 2.37 | 0.0806 | 2.236 | AKT2, PTPN11, RRAS, STAT5B, SYK |
| ERK/MAPK Signaling | 2.35 | 0.0463 | 2.53 | CRKL, EIF4E, HSPB1, MKNK1, NFATC1, PLA2G4A, PPP1R12A, PRKAR1A, RRAS, SRC |
| Estrogen Receptor Signaling | 2.34 | 0.0372 | 2.496 | AKT2, CACNB3, CDKN1A, DLG4, EGF, EIF2B4, EIF4E, FOXO1, FOXO3, NDUFB7, PLCB2, PPP1R12A, PRKAR1A, RRAS, SRC |
| Co-inhibition by BTLA | 2.34 | 0.0794 | 2.236 | PTPN11, PTPN6, STK24, STK39, STK4 |
| Semaphorin interactions | 2.31 | 0.0781 | 2.236 | ARHGAP35, ARHGEF12, MYH9, RHOC, RRAS |
| NGF Signaling | 2.3 | 0.0579 | 2.646 | AKT2, BAX, CHUK, IKBKG, MAP3K5, PTPN11, RRAS |
| KEAP1-NFE2L2 pathway | 2.29 | 0.0523 | 2.828 | AKT2, BACH1, CDKN1A, EIF2AK3, STK24, STK39, STK4, UFD1 |
| EPH-Ephrin signaling | 2.28 | 0.0652 | 2.449 | DNM1, GIT1, MYH9, SRC, VAV3, YES1 |
| Cardiac Hypertrophy Signaling (Enhanced) | 2.28 | 0.034 | 3 | AKT2, CACNB3, CD40LG, CHUK, EIF2B4, EIF4E, HDAC5, HDAC6, HSPB1, IKBKG, MAP2K6, MAP3K5, MKNK1, NFATC1, PLCB2, PRKAR1A, PRKG1, RRAS |
| B Cell Receptor Signaling | 2.28 | 0.0378 | 2.138 | AKT2, BCL2L1, CHUK, FOXO1, IKBKG, INPPL1, MAP2K6, MAP3K5, NFATC1, PTPN11, PTPN6, RRAS, SYK, VAV3 |
| Pyridoxal 5'-phosphate Salvage Pathway | 2.25 | 0.0758 | 2.236 | AKT2, EIF2AK2, GRK5, IRAK1, MAP2K6 |
| Pancreatic Adenocarcinoma Signaling | 2.24 | 0.0565 | 2 | AKT2, BCL2L1, CDKN1A, EGF, HDAC5, HDAC6, SMAD2 |
| GP6 Signaling Pathway | 2.24 | 0.0565 | 2 | AKT2, FYB1, GRAP2, LCP2, RASGRP2, SYK, VAV3 |
| April Mediated Signaling | 2.24 | 0.0952 | 2 | CHUK, IKBKG, NFATC1, TRAF2 |

| Ingenuity Canonical Pathways | -log (p-value) | Ratio | z-score | Molecules |
|---|---|---|---|---|
| Ovarian Cancer Signaling | 2.2 | 0.0506 | 2.236 | AKT2, AXIN1, EGF, HDAC5, HDAC6, PRKAR1A, RRAS, SRC |
| DAP12 interactions | 2.2 | 0.093 | 2 | GRAP2, LCP2, SYK, VAV3 |
| B Cell Activating Factor Signaling | 2.2 | 0.093 | 2 | CHUK, IKBKG, NFATC1, TRAF2 |
| RHO GTPases Activate WASPs and WAVEs | 2.16 | 0.0909 | 2 | SRC, WAS, WASF1, YES1 |
| Clathrin-mediated endocytosis | 2.15 | 0.0543 | 2.646 | DAB2, DNM1, EGF, FCHO2, GAK, LDLRAP1, REPS2 |
| GM-CSF Signaling | 2.14 | 0.0714 | 2.236 | AKT2, BCL2L1, PTPN11, RRAS, STAT5B |
| Pancreatic Secretion Signaling Pathway | 2.14 | 0.0431 | 2.53 | AKT2, ARHGEF1, ARHGEF12, CA13, EGF, PLCB2, PRKAR1A, SRC, STK39, STX16 |
| Interleukin-2 family signaling | 2.09 | 0.087 | 2 | INPPL1, PTPN6, STAT5B, SYK |
| iNOS Signaling | 2.06 | 0.0851 | 2 | CHUK, IKBKG, IRAK1, IRAK4 |
| Netrin Signaling | 2.02 | 0.0471 | 2.121 | ABLIM3, AKT2, CACNB3, NFATC1, PRKAR1A, PRKG1, STX16, VASP |
| Extra-nuclear estrogen signaling | 2.02 | 0.0667 | 2.236 | AKT2, EGF, FOXO3, HSPB1, SRC |
| Oxytocin Signaling Pathway | 2.01 | 0.0393 | 2.714 | AKT2, CACNB3, HSPB1, MYH9, NFATC1, PLA2G4A, PLCB2, PPP1R12A, PRKAR1A, PRKG1, RRAS |
| IGF-1 Signaling | 2.01 | 0.0571 | 2 | AKT2, FOXO1, FOXO3, PRKAR1A, PTPN11, RRAS |
| Co-stimulation by CD28 | 2 | 0.0816 | 2 | AKT2, GRAP2, SRC, YES1 |
| RHO GTPases Activate Formins | 1.96 | 0.05 | 2.646 | CKAP5, CLASP1, DAAM1, FMNL3, MRTFA, RHOC, SRC |
| Fc epsilon receptor (FCERI) signaling | 1.96 | 0.0429 | 3 | CHUK, GRAP2, IKBKG, LCP2, NFATC1, RASGRP2, SYK, TAB2, VAV3 |
| FAT10 Cancer Signaling Pathway | 1.94 | 0.0784 | 2 | AKT2, CHUK, IKBKG, SMAD2 |
| Signaling by FGFR1 | 1.91 | 0.0769 | 2 | MKNK1, PTPN11, SPRED2, SRC |

| Ingenuity Canonical Pathways | -log (p-value) | Ratio | z-score | Molecules |
|---|---|---|---|---|
| Apoptotic execution phase | 1.91 | 0.0769 | 2 | CASP3, CASP7, DBNL, STK24 |
| Cardiac Hypertrophy Signaling | 1.86 | 0.0391 | 2.333 | CACNB3, EIF2B4, EIF4E, HSPB1, MAP2K6, MAP3K5, PLCB2, PRKAR1A, RHOC, RRAS |
| RHOGDI Signaling | 1.85 | 0.0411 | -2.121 | ARHGAP35, ARHGEF1, ARHGEF12, MYH9, PIKFYVE, PPP1R12A, RHOC, SRC, WASF1 |
| Signaling by PTK6 | 1.83 | 0.0727 | 2 | ARHGAP35, DOK1, EGF, PTPN1 |
| Thrombin Signaling | 1.81 | 0.0405 | 2.828 | AKT2, ARHGEF1, ARHGEF12, EGF, PLCB2, PPP1R12A, RHOC, RRAS, SRC |
| Activation of NMDA receptors and postsynaptic events | 1.8 | 0.0588 | 2.236 | DLG4, GIT1, NRGN, PRKAR1A, SRC |
| LPS-stimulated MAPK Signaling | 1.8 | 0.0588 | 2 | CHUK, IKBKG, MAP2K6, MAP3K5, RRAS |
| Role of NFAT in Cardiac Hypertrophy | 1.79 | 0.0402 | 2.646 | AKT2, CACNB3, HDAC5, HDAC6, MAP2K6, PLCB2, PRKAR1A, RRAS, SRC |
| Neurexins and neuroligins | 1.77 | 0.0702 | 2 | DBNL, DLG4, HOMER2, PDLIM5 |
| PDGF Signaling | 1.76 | 0.0575 | 2.236 | CRKL, EIF2AK2, INPPL1, RRAS, SRC |
| Signaling by ERBB4 | 1.75 | 0.069 | 2 | DLG4, EGF, SRC, TAB2 |
| Signaling by PDGF | 1.75 | 0.069 | 2 | CRKL, PTPN11, SRC, STAT5B |
| Cancer Drug Resistance by Drug Efflux | 1.75 | 0.069 | 2 | AKT2, FOXO1, FOXO3, RRAS |
| ABRA Signaling Pathway | 1.74 | 0.0568 | 2.236 | ABLIM3, DMD, MRTFA, MYH9, SMTN |
| GADD45 Signaling | 1.72 | 0.0678 | 2 | CDKN1A, FOXO3, MAP2K6, SMAD2 |
| Adrenomedullin signaling pathway | 1.7 | 0.0412 | 2.646 | AKT2, BAX, CASP3, MAP2K6, PLCB2, PRKAR1A, PRKG1, RRAS |
| Sertoli Cell-Germ Cell Junction Signaling Pathway (Enhanced) | 1.7 | 0.0388 | 2.333 | AKT2, AXIN1, MAP2K6, MAP3K5, PRKAR1A, PRKG1, RRAS, SMAD2, SRC |
| Osteoarthritis Pathway | 1.7 | 0.0388 | 2.333 | CASP2, CASP3, CASP7, CHUK, FADD, FOXO3, SDC4, SMAD1, SMAD2 |

| Ingenuity Canonical Pathways | -log (p-value) | Ratio | z-score | Molecules |
|---|---|---|---|---|
| 3-phosphoinositide Degradation | 1.63 | 0.04 | 2.828 | INPP4B, INPPL1, PIKFYVE, PPP1R12A, PTPN1, PTPN11, PTPN6, THTPA |
| D-myo-inositol-5-phosphate Metabolism | 1.59 | 0.0392 | 2.646 | INPPL1, PIKFYVE, PLCB2, PPP1R12A, PTPN1, PTPN11, PTPN6, THTPA |
| Salvage Pathways of Pyrimidine Ribonucleotides | 1.58 | 0.0515 | 2.236 | AKT2, EIF2AK2, GRK5, IRAK1, MAP2K6 |
| Fcgamma receptor (FCGR) dependent phagocytosis | 1.57 | 0.0417 | 2.646 | MYH9, SRC, SYK, VAV3, WAS, WASF1, YES1 |
| HIF1α Signaling | 1.57 | 0.0388 | 2.828 | AKT2, CDKN1A, EGF, EIF4E, MAP2K6, MKNK1, NAA10, RRAS |
| Interferon gamma signaling | 1.56 | 0.051 | 2.236 | IRF3, PTPN1, PTPN11, PTPN6, TRIM25 |
| Protein folding | 1.54 | 0.0505 | 2.236 | KIFC3, RGS6, TBCA, TBCB, TBCC |
| Ribavirin ADME | 1.54 | 0.0597 | 2 | PNP, STK24, STK39, STK4 |
| Role of JAK1 and JAK3 in γc Cytokine Signaling | 1.54 | 0.0597 | 2 | PTPN11, RRAS, STAT5B, SYK |
| Cellular response to heat stress | 1.51 | 0.0495 | 2.236 | BAG5, DNAJB1, HDAC6, HSPB1, SERPINH1 |
| GPER1 signaling | 1.48 | 0.0485 | 2.236 | PRKAR1A, SRC, STK24, STK39, STK4 |
| Mouse Embryonic Stem Cell Pluripotency | 1.48 | 0.0485 | 2.236 | AKT2, AXIN1, PTPN11, RRAS, SMAD1 |
| PPAR Signaling | 1.47 | 0.0481 | -2.236 | CHUK, IKBKG, RRAS, STAT5B, TRAF2 |
| Role of Chondrocytes in Rheumatoid Arthritis Signaling Pathway | 1.46 | 0.0429 | 2.449 | CASP3, CHUK, FADD, FOXO3, IRAK1, IRAK4 |
| Cargo recognition for clathrin-mediated endocytosis | 1.45 | 0.0476 | 2.236 | DAB2, EGF, FCHO2, LDLRAP1, REPS2 |

| Ingenuity Canonical Pathways | -log (p-value) | Ratio | z-score | Molecules |
|---|---|---|---|---|
| Macrophage Classical Activation Signaling Pathway | 1.43 | 0.0389 | 2.646 | CD40LG, CHUK, IKBKG, IRAK1, IRAK4, IRF3, STAT2 |
| SAPK/JNK Signaling | 1.42 | 0.0331 | 3 | CRKL, FADD, HNRNPK, MAP3K5, MAP4K5, MAPK8IP3, MINK1, NFATC1, RRAS, TRAF2 |
| Signaling by VEGF | 1.42 | 0.0467 | 2.236 | AKT2, HSPB1, SRC, VAV3, WASF1 |
| Egg-Sperm Fusion Signaling Pathway | 1.42 | 0.0467 | 2.236 | AKT2, PRKAR1A, PTPN1, RAB27B, STX16 |
| Antioxidant Action of Vitamin C | 1.39 | 0.0459 | -2.236 | CHUK, IKBKG, PLA2G4A, PLCB2, STAT5B |
| IL-17 Signaling | 1.39 | 0.038 | 2.646 | AKT2, CD40LG, CXCL5, MAP2K6, RRAS, TAB2, TRAF2 |
| C-type lectin receptors (CLRs) | 1.38 | 0.0411 | 2.449 | CHUK, IKBKG, NFATC1, SRC, SYK, TAB2 |
| Estrogen-Dependent Breast Cancer Signaling | 1.37 | 0.0526 | 2 | AKT2, RRAS, SRC, STAT5B |
| Neurotrophin/TRK Signaling | 1.35 | 0.0519 | 2 | MAP2K6, MAP3K5, PTPN11, RRAS |
| Endothelin-1 Signaling | 1.35 | 0.0372 | 2.646 | CASP2, CASP3, CASP7, PLA2G4A, PLCB2, RRAS, SRC |
| Type II Diabetes Mellitus Signaling | 1.34 | 0.04 | 2.236 | AKT2, CACNB3, CHUK, IKBKG, MAP3K5, TRAF2 |
| D-myo-inositol (1, 4, 5, 6)-Tetrakisphosphate Biosynthesis | 1.34 | 0.037 | 2.449 | INPPL1, PIKFYVE, PPP1R12A, PTPN1, PTPN11, PTPN6, THTPA |
| D-myo-inositol (3, 4, 5, 6)-tetrakisphosphate Biosynthesis | 1.34 | 0.037 | 2.449 | INPPL1, PIKFYVE, PPP1R12A, PTPN1, PTPN11, PTPN6, THTPA |
| GNRH Signaling | 1.33 | 0.0368 | 2.449 | CACNB3, MAP2K6, MAP3K5, PLCB2, PRKAR1A, RRAS, SRC |
